# The switch between acute and persistent paramyxovirus infection caused by single amino acid substitutions in the RNA polymerase P subunit

**DOI:** 10.1371/journal.ppat.1007561

**Published:** 2019-02-11

**Authors:** Dan F. Young, Elizabeth B. Wignall-Fleming, David C. Busse, Matthew J. Pickin, Jacqueline Hankinson, Elizabeth M. Randall, Amy Tavendale, Andrew J. Davison, Douglas Lamont, John S. Tregoning, Steve Goodbourn, Richard E. Randall

**Affiliations:** 1 School of Biology, Centre for Biomolecular Sciences, BMS Building, North Haugh, University of St. Andrews, St. Andrews, Fife, United Kingdom; 2 MRC–University of Glasgow Centre for Virus Research, Glasgow, United Kingdom; 3 Mucosal Infection and Immunity Group, Section of Virology, Imperial College London, London, United Kingdom; 4 Institute for Infection and Immunity, St. George's, University of London, London, United Kingdom; 5 School of Life Sciences, University of Dundee, Dundee, United Kingdom; Johns Hopkins Bloomberg School of Public Health, UNITED STATES

## Abstract

Paramyxoviruses can establish persistent infections both *in vitro* and *in vivo*, some of which lead to chronic disease. However, little is known about the molecular events that contribute to the establishment of persistent infections by RNA viruses. Using parainfluenza virus type 5 (PIV5) as a model we show that phosphorylation of the P protein, which is a key component of the viral RNA polymerase complex, determines whether or not viral transcription and replication becomes repressed at late times after infection. If the virus becomes repressed, persistence is established, but if not, the infected cells die. We found that single amino acid changes at various positions within the P protein switched the infection phenotype from lytic to persistent. Lytic variants replicated to higher titres in mice than persistent variants and caused greater infiltration of immune cells into infected lungs but were cleared more rapidly. We propose that during the acute phases of viral infection *in vivo*, lytic variants of PIV5 will be selected but, as the adaptive immune response develops, variants in which viral replication can be repressed will be selected, leading to the establishment of prolonged, persistent infections. We suggest that similar selection processes may operate for other RNA viruses.

## Author summary

As well as causing acute infections that result in mild to serious disease, many RNA viruses can establish prolonged or persistent infections in some infected individuals, that occasionally lead to chronic or reactive disease. Little is known about the molecular mechanisms involved in the establishment of such infections. Using parainfluenza virus type 5 (PIV5) as a model, we show how lytic and persistent variants of the virus can be selected on the basis of single amino acid substitutions and propose that the selection of persistent variants as the adaptive immune response develops following an acute infection might be a mechanism these viruses have evolved to enhance their transmission rates. As well as being of fundamental interest, understanding the molecular basis by which RNA viruses establish persistent infections may improve our understanding of virus epidemiology (and hence improve the control of virus infections) and of virus:host interactions that influence the relationship between virus persistence and chronic/relapsing disease. Furthermore, the knowledge of how RNA viruses, such as PIV5, establish persistent infections may lead to improve vaccine design since vectors which can establish persistent infections may induce longer-lasting more robust immunity.

## Introduction

Paramyxoviruses are primarily known for the acute infections and associated diseases they cause, such as mumps, measles and respiratory illnesses. However, under certain conditions they can establish persistent (or prolonged) infections [1], which can be considered as infections that continue for longer than would be expected from a prototypical acute infection (2–3 weeks). For example, both immunocompromised and immunocompetent patients have been shown to shed parainfluenza virus (PIV) 2, 3 and 4 for weeks and even years after infection [1], whilst dogs infected with canine distemper virus can shed virus for months after initial infection [2]. Prolonged/persistent infections can lead to chronic diseases, for instance subacute sclerosing panencephalitis (SSPE) is associated with measles [3], postviral olfactory dysfunction is associated with PIV3 [4] and chronic kidney disease is associated with feline paramyxovirus [5, 6].

Little is understood of the molecular mechanisms by which paramyxoviruses establish and maintain persistent infections. Like other viruses, they must avoid elimination by the host immune response, while maintaining their genomes in at least some infected cells. Since it is highly probable that continual high-level viral replication in a cell will either directly kill the cell or lead to viral recognition and killing by innate and adaptive immune responses, it is likely that viral replication must be at least partially repressed in some cells in order for a virus to establish a persistent infection [7].

The interferon (IFN) response, and the ability of viruses to circumvent it, is also likely to play an important role in the establishment and maintenance of persistent infections. Thus, patients who have a defective ability to respond to type I IFN can become persistently infected with measles, mumps and rubella viruses following MMR vaccination with serious sequelae [8, 9]. On the other hand, we have suggested that the IFN response may also dampen down viral replication in some cells, thereby facilitating persistence [10, 11]. Using parainfluenza virus type 5 (PIV5) as a model, we show here that viral replication at late times after infection can also be repressed in an IFN-independent mechanism, thereby leading to the establishment of persistent infections.

PIV5 (previously known as simian virus 5; species *Mammalian rubulavirus 5*, genus *Rubulavirus*, family *Paramyxoviridae*) has been isolated from numerous mammals including, humans, primates, pigs, ungulates (cattle), dogs [12, 13] and lesser pandas [14]. There is also some evidence that cats, hamsters, rabbits and guinea pigs can be infected [15, 16]. The association of PIV5 with acute disease is often not obvious, although the virus causes kennel cough in canines [17] and may, or may not, cause acute respiratory symptoms in pigs [18, 19] and calves [20]. PIV5 can also cause unsuspected persistent infections of tissue culture cells, including the AGS line [21, 22], guinea pig kidney and mouse fibroblast cells [23], and is likely to establish persistent infections *in vivo* [24–26].

PIV5 has a non-segmented, negative-sense RNA genome of 15,246 nucleotides (nt) containing seven tandemly arranged genes, that encode eight proteins, flanked by 3’-leader and 5’-trailer sequences at the genome ends. From the 3’-leader sequence, the genome encodes the nucleocapsid protein (NP), V protein (V), phosphoprotein (P), matrix protein (M), fusion protein (F), small hydrophobic protein (SH), haemagglutinin-neuraminidase (HN), and the large protein (L). The genomic RNA is encapsidated by NP, forming a flexible helical nucleocapsid complex that is associated with the viral RNA-dependent RNA polymerase complex (vRdRP) consisting of L and P (extensively reviewed in ref [27]). Previous work has highlighted the importance of the phosphorylation of the P protein in regulating the activity of the vRdRP and influencing viral growth [28]. Furthermore, P plays a role in limiting the induction of host cell responses by influencing the fidelity of viral RNA synthesis [29]. The use of mass spectrometry has identified multiple sites on the P protein that can be phosphorylated, including serine residues at positions 36, 126, and 157 and a threonine residue at position 286 [30]. In addition, it has been shown that host cell Polo-like kinase 1 (PLK1) can phosphorylate a serine residue at position 308 [31]. Mutation of the serine residues to alanine residues at either position 157 or 308, thereby preventing phosphorylation at these residues, significantly enhanced the activity of the vRdRP in mini-genome assays and the replication of recombinant viruses that bear these mutations [31]. In contrast, phosphorylation of the threonine residue at position 286 may enhance viral replication, since mutating it to an alanine residue reduced vRdRP activity and viral growth [30]. Here we show that amino acid substitutions, found in natural isolates of PIV5, at residues 157 and 308, as well as at other sites, including some which cannot be phosphorylated, also influence the activity of the vRdRP in mini-genome assays and determine whether replication is or is not repressed at later times post infection leading to lytic or persistent phenotypes respectively. As only single nucleotide changes in all the wild-type isolates of PIV5 sequenced are predicted to convert them from lytic to persistent phenotypes, or visa versa, we propose that the selection of lytic or persistent variants, from a quasispecies population (defined as the mutant distributions that are generated upon replication of viruses in infected cells and organisms [32]), at early and late times after infection, respectively, may be a mechanism that PIV5, and some other RNA viruses, have evolved to increase their success of transmission.

## Results

### Switch-off of PIV5-W3 transcription and replication late in infection facilitates persistence

**Fig 1:**
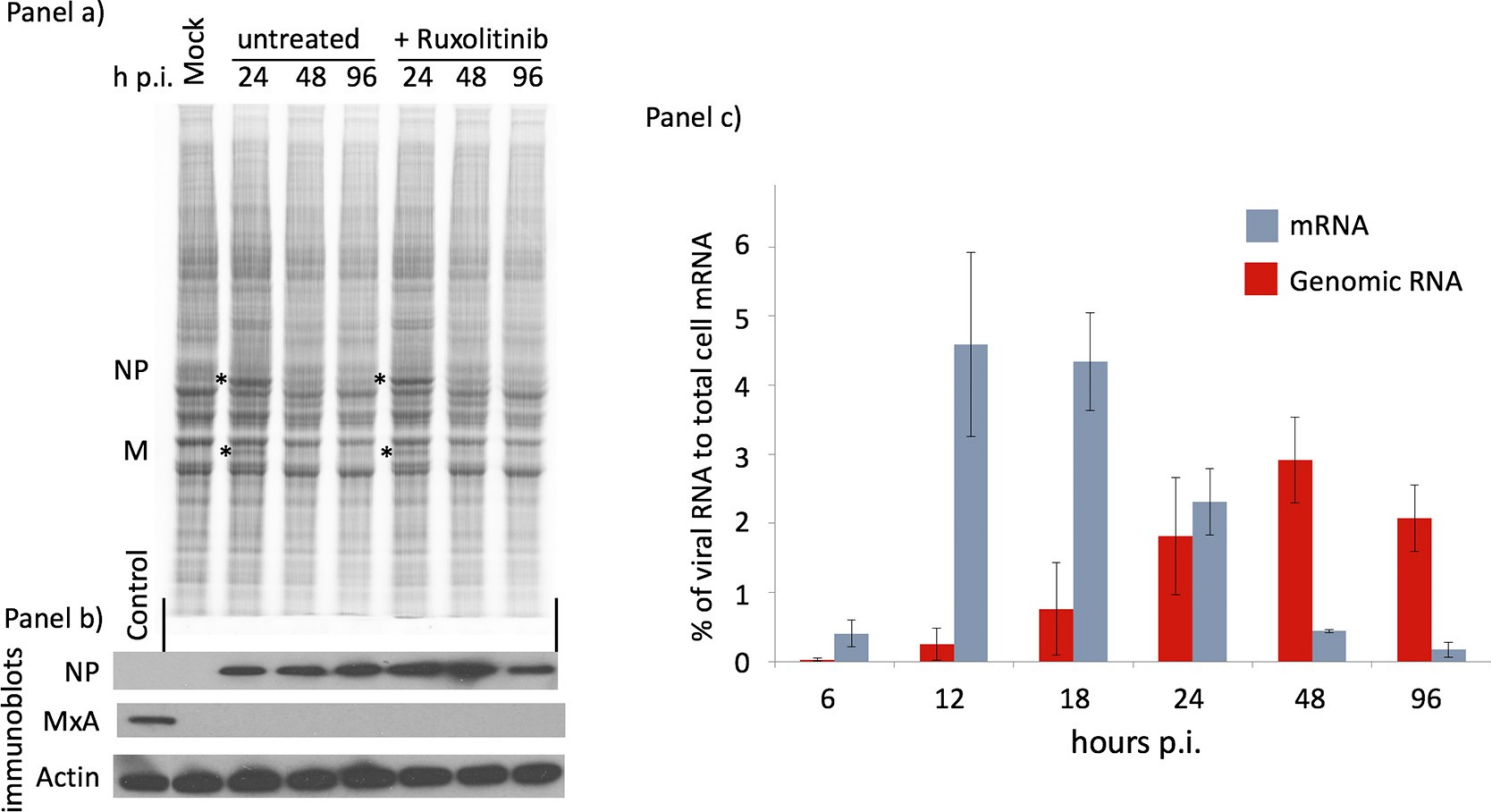
PIV5-W3 protein synthesis and transcription are repressed with time p.i. in an IFN-independent manner. Panel a) Monolayers of A549 cells were either mock infected or infected with PIV5 W3 at 10 pfu/cell in the presence or absence of Ruxolitinib (2μg/ml). At the times indicated the cells were metabolically labelled for 1h with [^35^S]-L-methionine. Polypeptides present in total cell extracts were separated by electrophoresis through a 4–12% SDS-PAG, and the labelled polypeptides visualized using a phosphorimager. The position of the NP and M polypeptides are indicated by asterisks (see S2 Fig). Note: PIV5 infection does not specifically switch off host cell protein synthesis and thus the relative levels of host cell protein synthesis in each track can be used as loading controls (S2 Fig). Panel b) The relative amounts of accumulated NP in the same samples shown in panel a) were visualized by immunoblot analysis. Also included as a control for IFN-induced MxA induction was a total cell lysate of mock-infected cells treated with IFN for 24h (control); immunoblotting for actin was used as loading controls. Panel c) Monolayers of A549 cells grown in 25cm flasks were infected with PIV5 W3 at 10 pfu/cell, RNA was extracted at 6, 12, 18, 24, 48 and 96h p.i. and subjected to total RNA sequencing following rRNA and mitochondrial RNA reduction. Directional sequence analysis was performed and the percentage of viral mRNA and genome reads were compared to the cellular reads at each time point. Error bars are based on three independent experiments. Note that although it is not possible to distinguish reads generated from viral mRNAs from those generated from antigenomes by directional sequencing, in the data presented here, because the proportion of antigenome reads cannot exceed that of the L gene extended over the whole genome, we can infer that the contribution of antigenome reads was <2% of the total mRNA/antigenome reads.

In contrast to infection with most strains of PIV5 held within our laboratory (see below), a high multiplicity of infection (moi) of A549, MRC5 or Vero cells with the PIV5-W3 isolate led to >90% of the cells surviving to become persistently infected; these infected cell-lines can be readily passaged (S1 Fig). We therefore decided to investigate the molecular events that lead to the establishment and maintenance of PIV5-W3 persistence in the expectation that this may lead to a better understanding of paramyxovirus persistence *in vivo*. Initially, we monitored the synthesis of viral proteins and the levels of viral mRNA and genomic RNAs following a high moi of A549 cells (Fig 1). Ongoing viral protein synthesis at various times post-infection (p.i.) was visualised by metabolically labelling A549 cells that had been infected with PIV5-W3 at a high moi, with [^35^S]-L-methionine for 1h (Fig 1, panel a). At 24h post infection (p.i.) NP and M were synthesised at sufficiently high levels to be clearly detectable above the background of cellular protein synthesis, which is not significantly repressed in PIV5-W3-infected cells. However, by 48 and 96h p.i., synthesis of NP and M had fallen below the levels that could be detected above the background of cellular protein synthesis. Immune precipitation analysis revealed that even by 36h p.i. there was an obvious reduction in the synthesis of all viral proteins (S2 Fig). In contrast, immunoblot analysis of the same samples showed that the relative levels of (accumulated) NP were slightly higher at 96h p.i. than at 24h (Fig 1, panel b), even though at 96h p.i. there was little, if any, *de novo* virus protein synthesis (Fig 1, panel a).

It was possible that the decrease in viral protein synthesis observed between 24 and 48h p.i. was due to an IFN-induced antiviral state within the infected cells. Although, this appeared unlikely because MxA, an IFN-induced protein, was not induced in the infected A549 cells (Fig 1, panel b), we monitored the kinetics of viral protein synthesis in cells that were infected in the presence of ruxolitinib (an inhibitor of JAK1 that blocks IFN signalling [33]). There were no changes in the switch-off kinetics in the presence of ruxolitinib (Fig 1, panel a). Furthermore, viral protein synthesis was also switched off with the same kinetics in A549/Npro cells which cannot produce IFN because BVDV-Npro targets IRF3 for proteasome-mediated degradation ([34]; S3 Fig), confirming that the decrease in virus protein synthesis observed was independent of the IFN response.

To investigate whether the observed switch-off of viral protein synthesis was due to inhibition of viral transcription, we used high-throughput sequencing (HTS) to quantitate the levels of viral mRNA and viral genomic RNA during infection. Maximal levels of viral transcription were observed between 12 and 18h p.i., at which times the amount of viral mRNA comprised almost 5% of total cellular mRNA (Fig 1 panel c). Thereafter, the amount of viral mRNA slowly declined such that by 96h p.i. it amounted to less than 0.2% of total cellular mRNA, thus indicating that it was the reduction in viral mRNA that was responsible for the observed switch-off of viral protein synthesis (note: although viral mRNA and antigenome sequences cannot be distinguished by directional sequencing, antigenome sequences contributed <2% of the total viral mRNA and antigenome reads, see Fig 1C legend). Levels of viral genomic RNA continued to increase until 48h p.i. (Fig 1, panel c). Strikingly, high levels of genomic RNA were also present at 96h p.i., by which time there was very little viral transcription occurring. Defective virus genomes (DVGs) were not detected by HTS at 96h p.i. (or in persistently infected cultures) suggesting that they do not play a role in the switch-off of viral transcription and protein synthesis (discussed in greater detail below).

**Fig 2:**
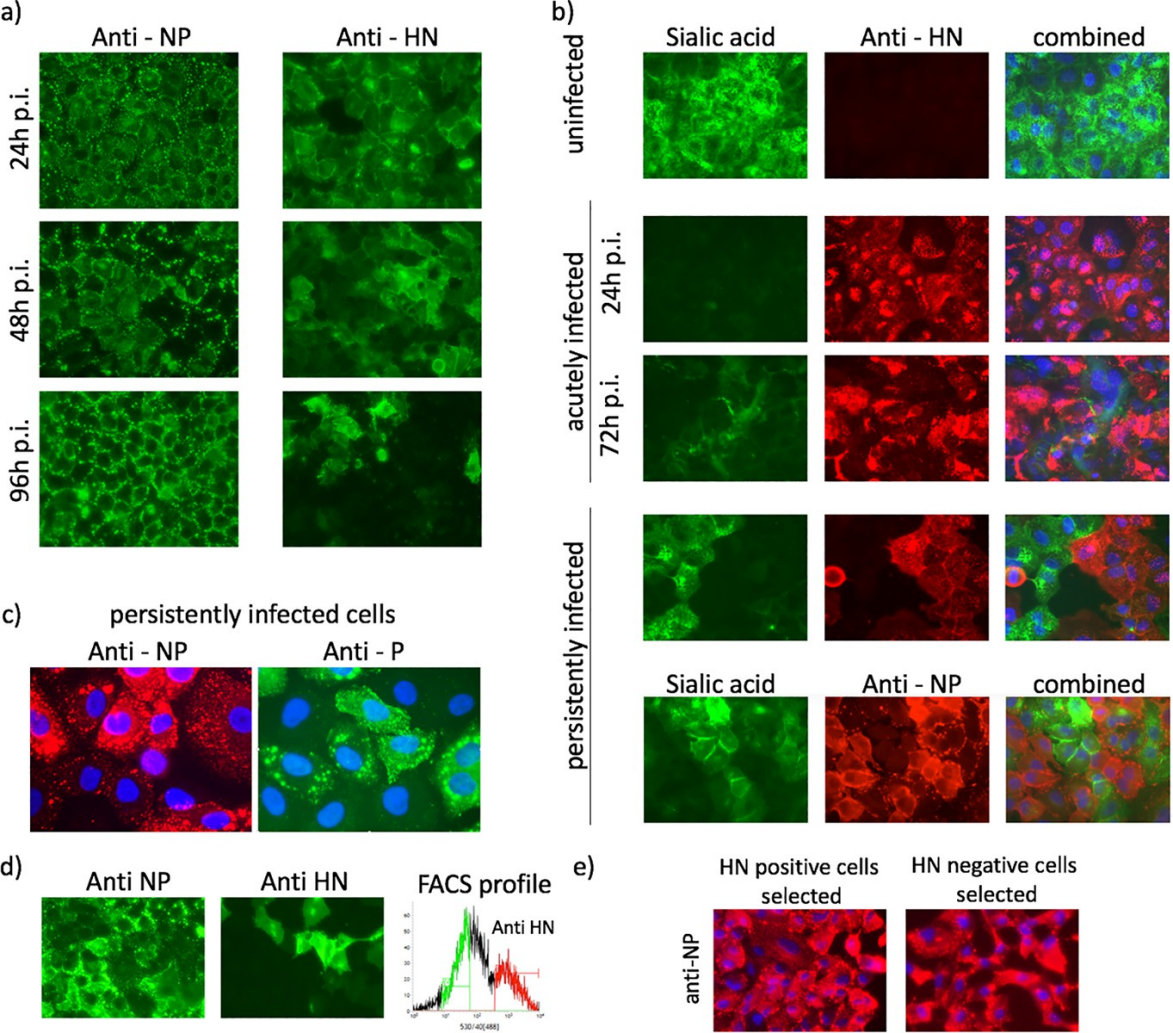
PIV5 fluxes between active and repressed states in persistently infected cells. Panel a) A549 cells grown on coverslips were infected with PIV5 W3 at 10 pfu per cell. At the indicated times p.i. the cells were fixed, permeabilized and stained with anti-NP and anti-HN monoclonal antibodies. Panel b) uninfected A549 cells or cells infected with PIV5 at 10 pfu/cell were fixed at 24 and 72h p.i. then stained for surface expression of sialic acid, or immunostained with an anti-HN monoclonal antibody. In addition, cells that were persistently infected with PIV5 (passage 3) were similarly stained, as well as being stained with an anti-NP monoclonal antibody. The nuclei were also visualised by staining the cells with DAPI (blue). Panel c) PIV5 (passage 3) persistently infected cells were immuostained with an anti-NP or anti-P mAb; the cells nuclei were also visualised by staining the cells with DAPI (blue). Note, some cells are strongly positive for NP or P, whilst others are only weakly positive. Panel d). A549 cells persistently infected with PIV5 W3 (p3) were fixed and immunostained for NP and HN and at the same time live cells were also immunostained for surface HN and the relative levels of HN expression measured by FACS analysis. Both HN-positive (red) and HN-negative (green) populations were separated as single cells into 96-well plates and colonies grown for 2 weeks in the presence of a pool of neutralizing monoclonal antibodies. Panel e) Examples of colonies of cells, derived from cells that had been FAC-sorted on the basis of whether they were or were not positive for HN, that were grown in 96-well plates were fixed and immunostained with anti-NP mAb, and counter-stained with DAPI. Cells were visualized using an EVOS microscope.

The switch-off of viral protein synthesis could also be inferred from immunofluorescence studies (Fig 2, panel a) aimed at visualising the presence within infected cells of HN (which has a half-life of 2.5 hours [35]) and NP (which has a half-life of days). At 24h p.i. all cells were strongly positive for both NP and HN. However, by 96h p.i., while all the cells remained positive for NP, less than 50% of the cells were also positive for HN, and many of those were only weakly positive (Fig 2, panel a). Since HN possesses neuraminidase activity, the levels of HN expression at later times p.i. could also be inferred by staining cells for the presence of sialic acid. At 24h p.i. none of the infected cells expressed detectable amounts of sialic acid on their surface (Fig 2, panel b). However, by 72h p.i. some cells were positive for sialic acid. The fact that a high proportion of cells were negative for HN, and positive for sialic acid, strongly suggests that there was little or no ongoing viral protein synthesis in these cells at late times p.i.. These results demonstrate a degree of cellular asynchrony in the relative levels of viral gene expression at late times p.i..

Cells persistently infected with PIV5-W3 grew slightly slower than uninfected cells for the first couple of passages but by passage 3 (p3) replicated as fast as uninfected cells and showed few visual signs of being infected. Immunostaining of persistently infected cells at p3 showed that whilst all the cells were infected there was heterogeneity in expression of the HN protein (Fig 2, panel b). Thus, some cells were positive for HN and negative for sialic acid, and others were negative for HN and positive for sialic acid. All cells were positive for NP and P expression, although the amount of NP and P present in the cells varied considerably (Fig 2, panels b and c). In general, cells that were strongly positive for NP (and P) were negative for sialic acid, and those that were weakly positive for NP (and P) were positive for sialic acid. HTS of the cells at 96 h p.i., and of persistently infected cells, showed that viral mRNA constituted less than 0.2% of the total RNA (Fig 1, panel c). This level of viral mRNA characterised the population of cells as a whole but, given the heterogeneity of virus expression, the levels of viral mRNA must have varied considerably among cells within the persistently infected population. These results therefore strongly suggest that within the persistently infected population as a whole, active viral transcription was occurring in some cells (HN-positive cells) but was largely, if not completely, repressed in others (HN-negative cells).

### Infectious PIV5-W3 can be recovered from persistently infected cells in which viral transcription is repressed

We next investigated whether infectious virus could be rescued from cells in which viral transcription and replication were repressed. Persistently infected cells at passage 3 were stained for surface expression of the HN protein, and FACS was used to sort HN-positive and HN-negative cells into individual wells of 96 well plates (Fig 2, panel d). Colonies from each population were grown out in the presence, or absence, of a pool of PIV5-neutralizing antibodies to prevent viral spread between cells. Immunofluorescence showed that all the colonies remained infected (Fig 2, panel e), regardless of whether the sorted cells were derived from HN-positive or HN-negative cells, and whether or not they had been cultured in the presence or absence of neutralizing antibody. Furthermore, upon removal of the medium containing neutralizing antibody, infectious virus was recovered from all colonies tested. The fact that all the cells remained positive for NP in the presence of high titres of neutralizing antibody demonstrates that the cells remained infected as they divided, and that the production of infectious virus was not required for the maintenance of persistence within the colonies.

### Phosphorylation of the PIV5-W3 P protein is responsible for the switch-off of viral transcription and replication

**Fig 3:**
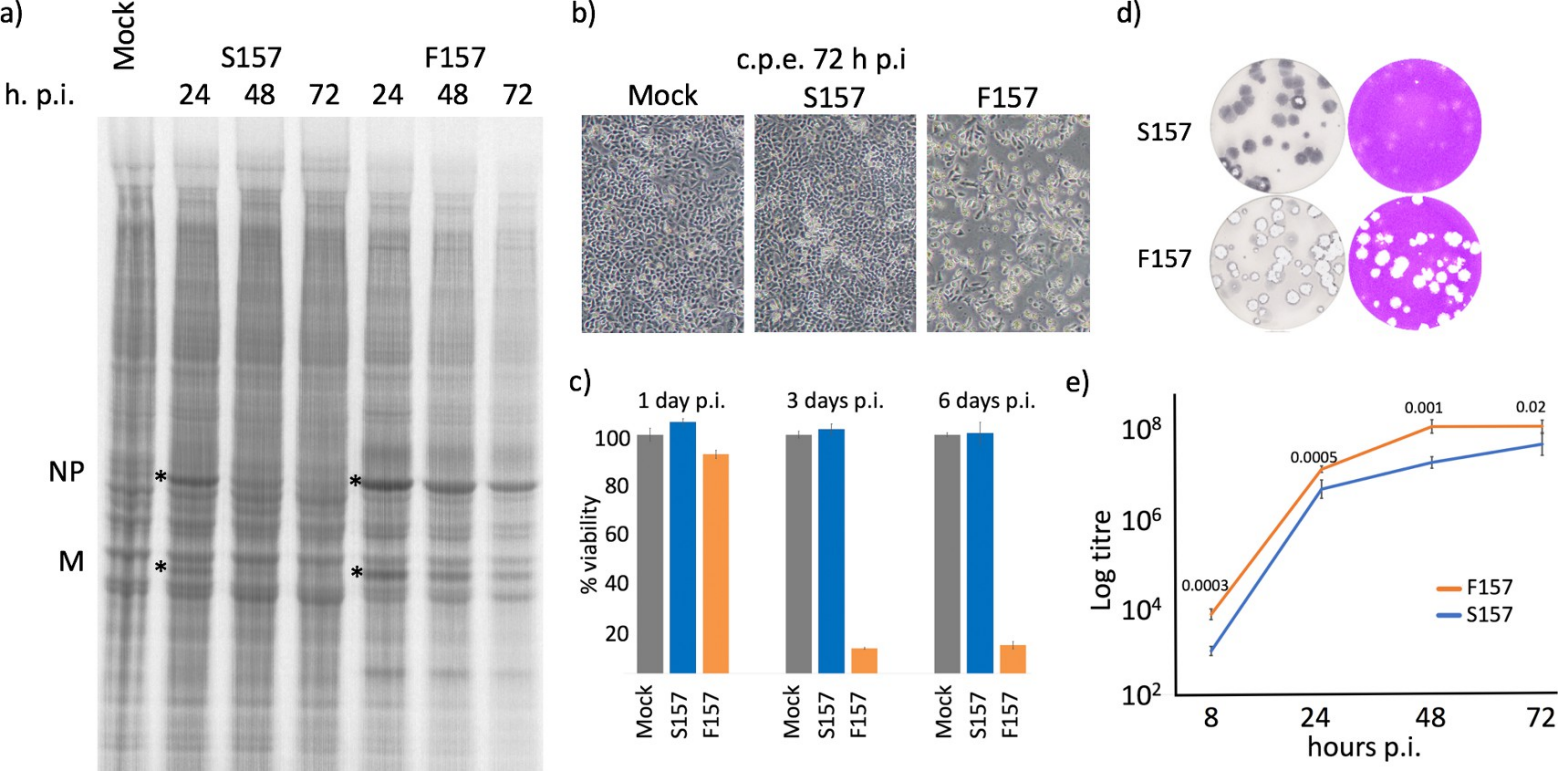
Single amino acid (nucleotide) change determines whether PIV5 has an acute or persistent phenotype. Panel a) Monolayers of A549 cells were either mock infected or infected with rPIV5-W3:P(S157) or rPIV5-W3:P(F157) at 10 pfu/cell. At the times indicated the cells were metabolically labelled for 1h with [^35^S]-L-methionine. Polypeptides present in the total cell extracts were separated by electrophoresis through a 4–12% SDS-PAG, and the labelled polypeptides visualized using a phosphorimager. Panel b) At the time of labelling images of the monolayers were taken using a phase contrast microscope. Note the lack of a cpe at 72h p.i. in cells infected the rPIV5-W3:P(S157) and almost complete death in cells infected with rPIV5-W3:P(F157). Panel c) Monolayers of A549 cells were either mock infected or infected with rPIV5-W3:P(S157) or rPIV5-W3:P(F157) at 10 pfu/cell and at the times indicated washed once and the % cell viability monitored using PrestoBlue cell viability reagent. Data shown represents mean values (n = 6 replicates; error bars = SD). Panel d) Plaques of rPIV5-W3:P(S157) and rPIV5-W3:P(F157) formed on monolayers of A549 at 4 days p.i. Plaques were either visualised by immunostaining the monolayers or staining the monolayers with crystal violet. Panel e) One step growth curve of A549 cells infected with either rPIV5-W3:P(S157) or rPIV5-W3:P(F157). Values for all times points were statistically significant (the P values in a T-test for each time point are shown). Data shown represents mean values (n = 6 replicates; error bars = SD) and the figure is representative of 3 independent experiments.

Previous work has shown that phosphorylation of PIV5 P regulates the activity of the vRdRP and that phosphorylation of the serine (S) residue at position 157 (S157) of the P protein plays a role in down-regulating viral gene expression [31, 36]. To determine whether S157 plays a critical role in the switch-off of viral transcription and replication, and in the establishment of persistence, we generated a recombinant virus, rPIV5-W3:P(F157), in which the serine residue at position 157 was replaced by a phenylalanine (F) residue in the PIV5-W3 backbone. The F substitution was chosen because several strains of PIV5 have this residue at position 157. Sequence analysis of rPIV5-W3:P(F157) confirmed that this was the only amino acid substitution in the recombinant virus. Following infection with rPIV5-W3:P(S157) at high moi, switch-off of viral protein synthesis occurred 24 and 72h p.i., and >95% of the cells survived the infection (Fig 3, panels a and b). In striking contrast, no detectable switch-off of viral transcription or replication occurred in cells infected with rPIV5-W3:P(F157), and by 72 h p.i. ~90% of infected cells had died (Fig 3, panels a, b and c). Furthermore, rPIV5-W3:P(S157) generated poorly defined plaques, which needed to be immunostained for clear visualisation, whereas plaques produced by rPIV5-W3:P(F157) were easily visualised by crystal violet staining because the cells within the plaques died, leaving obvious holes in the monolayer (Fig 3, panel d). Significantly, in single-step growth curves, rPIV5-W3:P(F157) grew more rapidly and to higher titres than rPIV5-W3:P(S157) (Fig 3, panel e).

It has been shown previously that a cellular kinase, Polo-like kinase 1 (PLK1), interacts with the P protein through the phosphorylated S157 residue and phosphorylates other sites on the P protein, including S308; phosphorylation at either of these sites reduces virus transcription [31]. We used mass spectrometry to compare the phosphorylation of the P protein in cells infected with rPIV5-W3:P(S157) and rPIV5-W3:P(F157) (S4 Fig). These results confirmed that S157 and S308 were phosphorylated in cells infected with rPIV5-W3:P(S157). Despite identifying multiple other phosphorylation sites on P in this way, we did not identify any sites, other than S157, that were phosphorylated on rPIV5-W3:P(S157) but not on rPIV5-W3:P(F157), and vice versa, including S308 (S4 Fig). However, we could not rule out the possibility that the relative levels of phosphorylation at the different sites did not vary significantly between rPIV5-W3:P(S157) and rPIV5-W3:P(F157). In a previous study, Sun et al [31], showed that a PLK1 kinase inhibitor (BI2536) increased PIV5-W3 gene expression in infected cells at 18–20h p.i.. Therefore, we tested whether BI2536 treatment prevented or delayed the switch-off of rPIV5-W3:P(S157) protein synthesis (S5 Fig). BI2536 had no discernible effect on the switch-off of viral protein synthesis, or the ability of rPIV5-W3:P(S157) to establish a persistent infection, suggesting that PLK1 is not the only cellular kinase that can phosphorylate serine-157 and inhibit viral gene expression.

### Amino acid residues in PIV5-W3 P other than that at position 157 can determine whether viral transcription and replication are repressed at late times p.i

**Fig 4:**
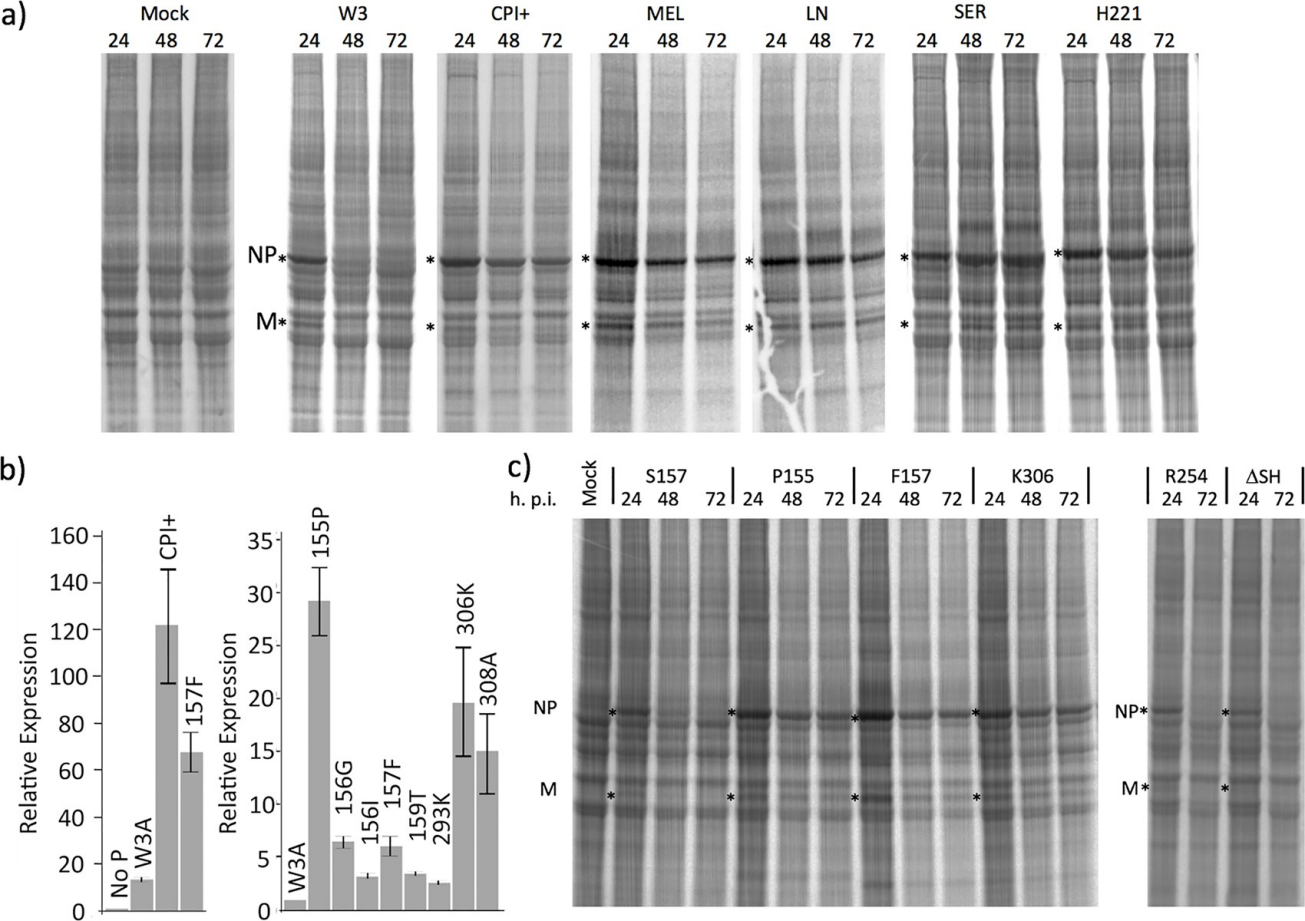
Single amino acid substitutions at multiple site on P influence the activity of the vRdRP and whether virus replication is repressed at late time after infection. Panel a). Monolayers of A549 cells were mock infected, or infected with the W3, CPI+, MEL, LN, SER or H221 strains of PIV5 at 10 pfu/cell. At the times indicated the cells were metabolically labelled for 1h with [^35^S]-L-methionine. Polypeptides present in the total cell extracts were separated by electrophoresis through a 4–12% SDS-PAG, and the labelled polypeptides visualized using a phosphorimager. The positions of the NP and M polypeptides are indicated by asterisks. Panel b) 293 cells were transfected with the minigenome plasmid pPIV5MG-Fluc.ter, helper plasmids expressing PIV5-NP, -P and -L, a plasmid expressing codon-optimised T7 RNA polymerase and a plasmid directing β-galactosidase expression as described in Materials and Methods. Transient transfections were left for 40 hours before harvesting, then luciferase and β-galactosidase expression assays were carried out. Left-hand panel; P of W3 was compared to P of CPI+ and P of the S157F variant of W3. The observed stimulations were compared to the result seen in the absence of P (set at 1.0). Right-hand panel; P of W3 was compared to the indicated variants of P; the observed stimulations were compared to the stimulation seen with P of W3 (set at 1.0). Panel c). Monolayers of A549 cells were either mock infected or infected with rPIV5-W3:P(S157), rPIV5-W3:P(P155), rPIV5-W3:P(F157), rPIV5-W3:P(K306), rPIV5-W3:P(R254) and rPIV5-W3:ΔSH at 10 pfu/cell. At the times indicated the cells were metabolically labelled for 1h with [^35^S]-L-methionine. Polypeptides present in the total cell extracts were separated by electrophoresis through a 4–12% SDS-PAG, and the labelled polypeptides visualized using a phosphorimager.

Having established that both transcription and replication of the W3 strain of PIV5 are significantly down-regulated within 48h, we next investigated whether this was the case for other PIV5 strains. A549 cells were infected at high multiplicity with the W3, CPI+, MEL, LN, SER and H221 strains of PIV5 and were metabolically labelled at various times p.i. with [^35^S]-L-methionine. Expression of NP and M proteins was repressed with time in cells infected with PIV5-W3, but no obvious switch-off of viral protein synthesis was observed for the other strains of PIV5 (Fig 4, panel a). HTS confirmed that high levels of virus protein synthesis at late times p.i. in CPI+-infected cells were because virus transcription was not repressed (S6 Fig, panel a). These studies also showed that the maximal levels of viral mRNA were significantly higher in CPI+ and rPIV5-W3:P(F157), approximately 17% and 13% respectively (S6 Fig, panel a and b), than in rPIV5-W3:P(S157)-infected cells (approximately 5%; Fig 1, panel b).

**Fig 5:**
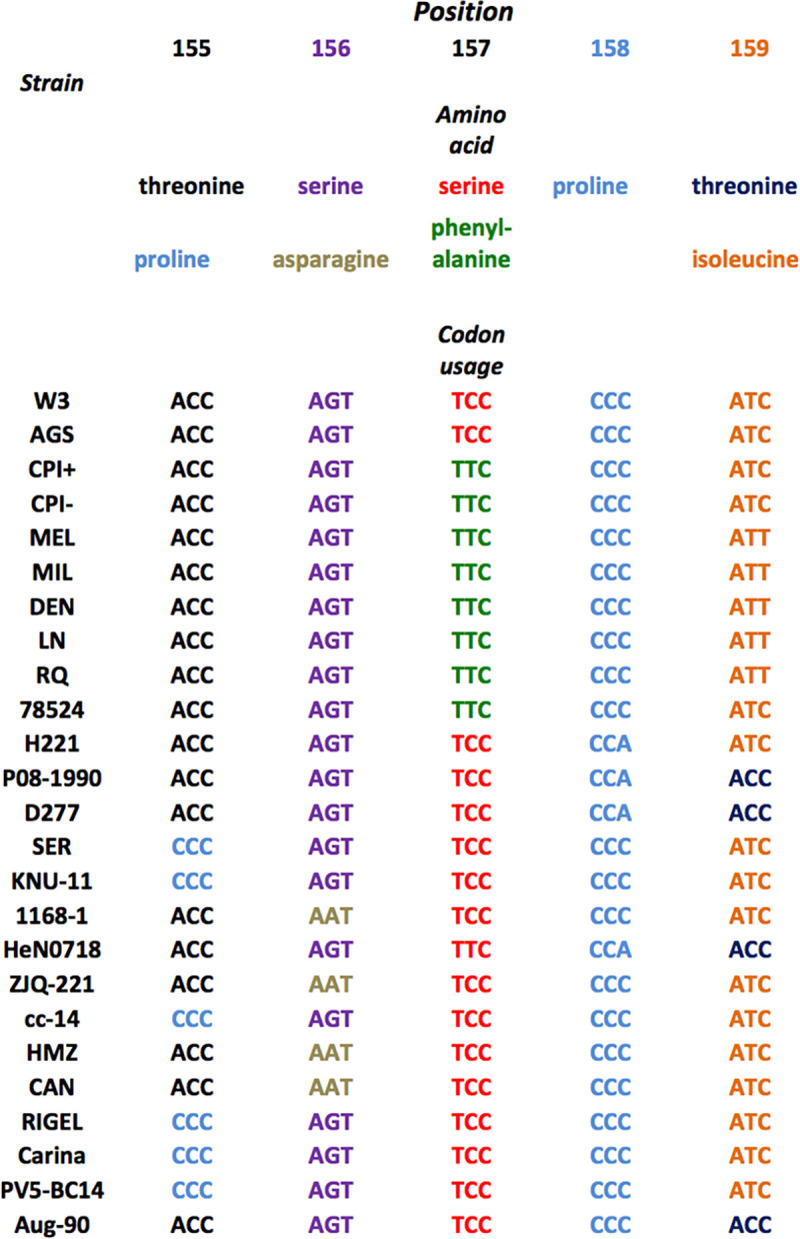
Amino acid and codon usage of strains of PIV5 in the NCBI database between amino acids 155–159 in P. Colours of the indicated amino acids are the same colours as for their respective codons.

A comparison of PIV5 P protein sequences published in GenBank strains revealed that CPI+, MEL and LN have F157 (Fig 5), consistent with their failure to shut-down expression as observed with rPIV5-W3(F157). However, we had expected that viral protein synthesis would be repressed at late times p.i. with H221 and SER because they have a serine at residue 157, but it was not. There are three amino acid sequence differences in P between PIV5-W3 and PIV5-SER (S69L, T155P and T293K) [13]; strikingly, T155P is in close proximity to S157. There are four amino acid differences in P between PIV5-W3 and PIV5-H221 (V226M, T293K, N306K and I381D); N306K is in close proximity to S308. We next checked whether P155, K306, and other amino acid changes around S157, have a direct effect on vRdRP activity by using a minigenome system. We initially compared the ability of P from PIV5-W3 (S157) and PIV5-CPI+ to stimulate viral RNA synthesis (Fig 4, panel b). In agreement with previously published data [36], P from PIV5-CPI+ was considerably more active than that derived from PIV5-W3. As predicted, substituting S for F at position 157 stimulated vRdRP activity (Fig 4, panel b). We next examined the phenotypes of other changes in the W3 P protein. The T155P substitution (as observed in PIV5-SER) significantly enhanced the activity of PIV5-W3 P in the minigenome assay (Fig 4, panel b). We also noted stimulatory effects of amino acid substitutions at positions 156 and 159. The N306K substitution (observed in PIV5-H221) and a substitution at amino acid 308 (S308A) also significantly enhanced P activity. In contrast, the T293K substitution (observed in both PIV5-SER and -H221) had only a small effect. These data show directly that the potential for phosphorylation in two motifs TSSPI (residues 155–159) and NDS (residues 306–308) represent targets for the negative regulation of P activity.

To further test the effect of changes in P on the gene expression profile of W3, we generated recombinant viruses with a T to P substitution at position 155, rPIV5-W3:P(P155), or an N to K substitution at position 306, rPIV5-W3:P(K306). Strikingly, both rPIV5-W3:P(P155) and rPIV5-W3:P(K306) behaved similarly to rPIV5-W3:P(F157), in that viral protein synthesis was not inhibited at late times p.i. (Fig 4, panel c), and viral infection resulted in increased cell death. In contrast, substituting a lysine for an arginine residue at position 254 (rPIV5-W3:P(R254)), which is part of a putative sumoylation site [37], had no effect on the switch-off of PIV5-W3 transcription, neither did deletion of the SH gene (Fig 4, panel c). These results demonstrate that single amino acid substitutions at multiple sites within P (observed in natural isolates of PIV5) can switch PIV5 from a virus with a persistent phenotype to one with a lytic phenotype.

### Single nucleotide substitutions in all sequenced PIV5 strains are sufficient to convert them from lytic to persistent phenotypes

Having shown that the repression of viral transcription and replication, and the establishment of persistence, depends on the integrity of the TSSPI motif at residues 155–159 in PIV5-W3, we compared all the PIV5 P protein and gene sequences available in the GenBank database (Fig 5). It was striking that in all strains residue 155 was either threonine or proline, residue 156 was either serine or asparagine, residue 157 was either serine or phenylalanine, and residue 159 was either threonine or isoleucine. Residue 158 (proline) was invariant. No strain had more than one difference from W3 in this region (e.g. none had both a proline at residue 155 and a phenylalanine at residue 157), and codon redundancy at these residues was such that a single nucleotide substitution was sufficient to change the virus from one with a predicted lytic to a W3-like persistent phenotype. Similarly to PIV5-W3, some strains had threonine at residue 155 and serine at residue 157 but differed from PIV-W3 sequence at neighbouring residues that increased vRdRP activity in the minigenome assays (e.g. residue 156 could be serine or asparagine, and residue 159 isoleucine or threonine; Fig 4, panel b). However, again only one nucleotide substitution was required to convert the sequence of P in these strains to that of PIV5-W3. It is also notable that PIV5-H221 was the only strain with lysine instead of asparagine at residue 306, and again codon redundancy ensured that a single nucleotide substitution was sufficient to convert it to a PIV-W3 phenotype.

### Defective virus genomes and PIV5 persistence

Defective virus genomes (DVGs) have been shown to play a role in the establishment and maintenance of persistent infections of tissue culture cells by many positive and negative sense RNA viruses [38]. We therefore used HTS to determine whether DVGs may play a role in the establishment of persistence with PIV5-W3. Firstly, we showed that HTS both of purified nucleocapsids and of total cell RNA (following the physical removal of ribosomal and mitochondrial RNA) could be used to successfully detect the presence of DVGs (S7 Fig). From this analysis we determined that we would detect any DVGs if their breakpoint sequences contributed more than 0.02% of genomic sequences, or if the DVG contributed more than 0.2% (or even less, see below) of the total DVG and non-defective genomes. Using this approach, no DVGs were detected, either in purified nucleocapsids or in total cell RNA, isolated from passage 3 persistently infected PIV5-W3 cells. HTS both of nucleocapsid RNA and total cell RNA extracted from p3 persistently infected cells also revealed that there were no changes in the consensus sequence of PIV5-W3 in the persistently infected cells.

Although, approximately 80% of cells die by 3 days p.i. following infection with CPI+ (as determined by measuring cell viability using PrestoBlue as described in Fig 3), some cells survive and, with difficulty, it is possible to establish a persistently infected cell-line from these surviving cells. This necessitated that the surviving cells be cultured for many weeks without sub-culturing, replacing the culture medium regularly. Eventually the surviving cells began to grow and could be passaged. However, even then they continued to grow poorly and showed obvious signs of virus cytopathic effects within the monolayers. High levels of DVGs (the ratio of DVGs to non-defective genomes was 1.7:1, S7 Fig) were detected in cells persistently infected with CPI+, suggesting that they may play a role in the establishment of persistent infections under these conditions. Also, in marked contrast to cells persistently infected with PIV5-W3 in which the amount of viral mRNA was less than 0.2% of total cellular mRNA, the levels of CPI+ mRNA in persistently infected cells was significantly higher, approximately 6% of total cellular mRNA. Furthermore, several polymorphic mutations were identified in the CPI+ persistently infected cell-lines but all of these, except for one, were synonymous mutations. The exception was located at position 13093 of the genome, an A to T change, resulting in a phenylalanine to leucine substitution in L, that was present in 17% of the reads, but the biological significance of this is unclear.

### rPIV5-W3:P(F157) replicates better in mice than rPIV5-W3:P(S157), causes more cellular infiltration into infected lungs, but is cleared more rapidly

**Fig 6:**
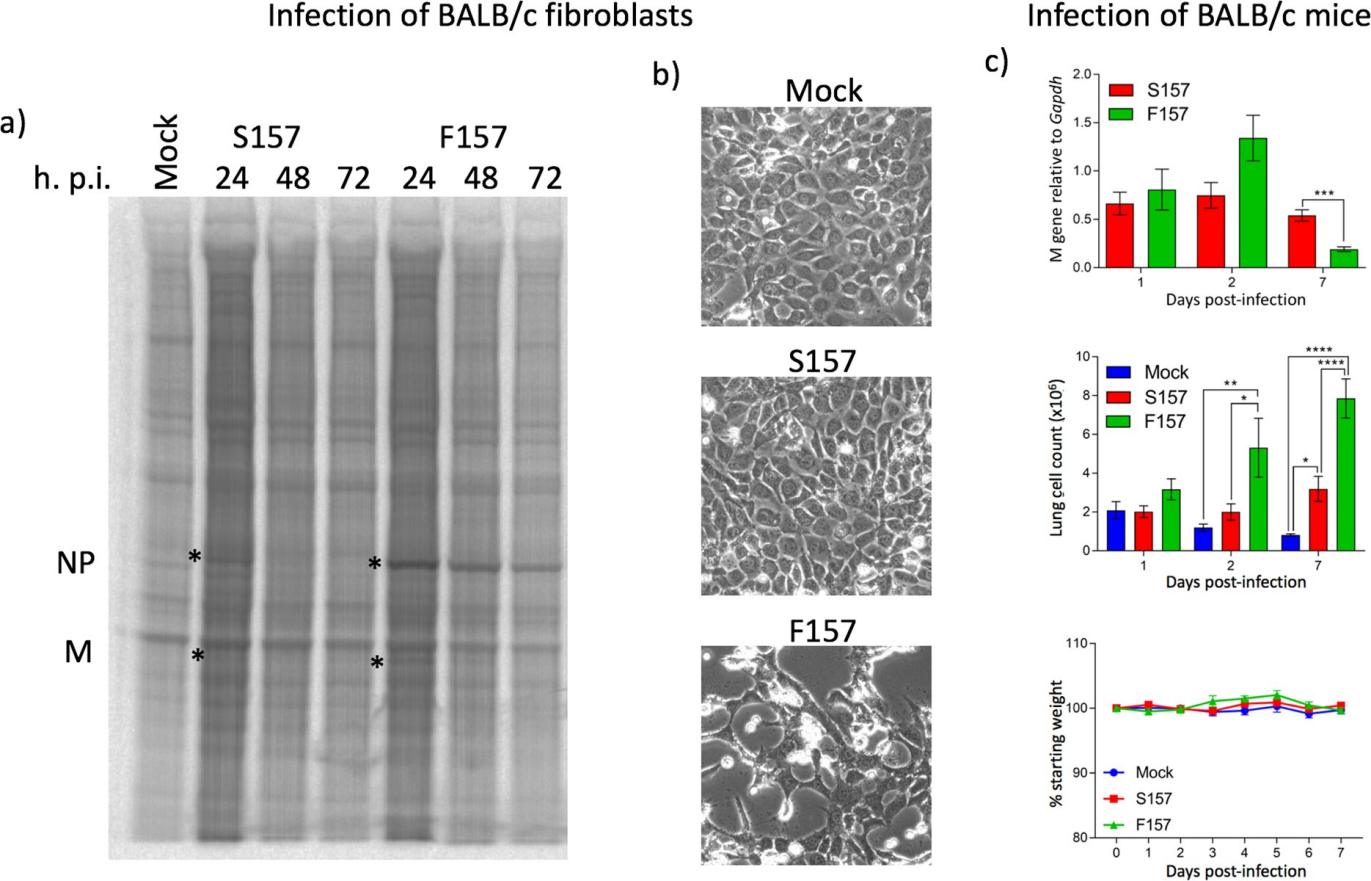
Lytic variants of PIV5 grow to higher titres in mice and induce more inflammation but are cleared more rapidly than persistent variants. Panel a) Monolayers of BALB/c fibroblast cells were either mock infected or infected with rPIV5-W3:P(S157) or rPIV5-W3:P(F157) at 10 pfu/cell. At the times indicated cells were metabolically labelled for 1h with [^35^S]-L-methionine. Polypeptides present in the total cell extracts were separated by electrophoresis through a 4–12% SDS-PAG, and the labelled polypeptides visualized using a phosphorimager. Panel b) At the time of labelling at 72h p.i. images of the monolayers were taken using a phase contrast microscope. Note the lack of a cpe at 72h p.i. in cells infected with the rPIV5-W3:P(S157) in contrast to cells infected with rPIV5-W3:P(F157). Panel c) BALB/c mice were infected with either rPIV5-W3:P(S157) or rPIV5-W3:P(F157). At 1, 2, and 7 days p.i. 5 mice were sacrificed, and the amount of virus present estimated by quantitative PCR (top panel) and the lung cell count measured (middle panel); their body weight was also measured daily until 7 days p.i. (bottom panel).

We wished to investigate whether the phenotypic differences observed between rPIV5-W3:P(S157) and rPIV5-W3:P(F157) were reflected in differences in their biological properties in a mouse model system. However, first, and in agreement with our previously published data [39], we demonstrated that, as observed in A549 cells, rPIV5-W3:P(S157) protein synthesis was switched off in BALB/c fibroblasts and that the cells survived the infection. In contrast, rPIV5-W3:P(F157) protein synthesis was not switched off in murine fibroblasts by 72 h p.i. and most of the cells died following infection (Fig 6, panels a and b).

To determine whether rPIV5-W3:P(S157) and rPIV5-W3:P(F157) had different biological properties *in vivo*, BALB/c mice were infected intranasally with rPIV5-W3:P(S157) or rPIV5-W3:P(F157) and sacrificed at 1, 2, and 7 days p.i., and the amount of virus present in the lungs was estimated by quantitative PCR (Fig 6, panel c). In addition, the amount of inflammation at the time of sacrifice was assessed by measuring the number of cells in the lungs (Fig 6, panel c) [40]. These results showed that, although rPIV5-W3:P(F157) had replicated to higher titres than rPIV5-W3:P(S157) by 2 days p.i., it was cleared more rapidly. Thus, by 7 days p.i. there was significantly less rPIV5-W3:P(F157) present in the lungs than rPIV5-W3:P(S157) (p<0.001). Interestingly, the amount of rPIV5-W3:P(S157) present in the lungs at 7 days p.i. was similar to that observed at 1 and 2 days p.i.. There were significantly more cells in the lungs after rPIV5-W3:P(F157) infection at days 2 (p<0.05) and 7 (p<0.01) p.i. than rPIV5-W3:P(S157), indicating greater inflammation [40]. However, neither virus caused overt disease as measured by weight loss (Fig 6, panel c).

## Discussion

Within-host RNA viral persistence has many potential consequences for both virus and the host [7]. For example, persistently infected individuals may act as viral reservoirs within host communities, and persistent infections may be important in the development of long-lasting protective immunity. However, little is known about the molecular mechanisms by which RNA viruses establish such infections. In part this may be because, unlike the better understood situations with DNA viruses or retroviruses, and despite the examples of hepatitis C virus and bornaviruses, it is often difficult to determine whether certain RNA viruses have evolved specific molecular mechanisms to establish and maintain persistent infections. To establish such infections within a host following lytic infection, it is likely that viral replication must be repressed in at least some cells in order either to prevent viral replication from killing the cell or to avoid the infected cell being eliminated by innate and adaptive immune responses [7]. In the case of paramyxoviruses and other members of the order *Mononegavirales*, and given their general mode of replication, it is not obvious how viral replication could be specifically repressed in order to facilitate virus persistence.

Using PIV5 as a model, we report that viral transcription and replication can be repressed by phosphorylation of P, resulting in the establishment of persistently infected cell cultures (without the need for the presence of DVGs) in which the virus can flux between active and repressed states within individual cells. Since the consensus genome sequence of PIV5-W3 does not change in persistently infected cells we suggest that the amount of P (Fig 2, panel c), as well as its level of phosphorylation, varies heterogeneously over time within persistently infected cells and it is this which determines whether the virus is active or repressed within individually infected cells. We also speculate that *in vivo*, depending on the status of the adaptive immune response, variants with lytic or persistent phenotypes will be selected for or against. Since the quasispecies nature of RNA viruses will generate a cloud of virus mutants *in vivo* [32, 41, 42] as the virus spreads from cell to cell, during acute phases of infection rapidly replicating PIV5 variants will be selected for. However, eventually cells that continuously synthesize high levels of viral proteins will be efficiently killed by cytotoxic T cells and possibly ADCC (antibody dependent cell cytotoxicity). Consequently, as the adaptive immune response develops, variants whose replication may be repressed, thus avoiding cell killing, will be selected, leading to the establishment of persistence. Since virus can be reactivated from cells in which it has been repressed, it is likely that small amounts of infectious virus will continuously be produced in persistently infected individuals perhaps as the immune response waxes and wanes. If such variants are transmitted to a new host again initially rapidly replicating variants will have a selective advantage, setting up a cycle of alternative selection of acute vs persistent variants in at least some infected individuals. Given that single amino acid (nucleotide) changes can determine whether a particular PIV5 variant has a lytic or persistent phenotype this mechanism may have evolved to allow PIV5 to establish both productive acute infections as well as persistent infections, thereby potentially increasing its chances of transmission [43]. For similar reasons, it is possible that other RNA viruses may have evolved analogous mechanisms in which single amino acid (nucleotide) changes can determine whether a particular variant has a lytic or persistent phenotype.

Our results support and extend those published by the He group showing that phosphorylation of S157 and S308 on P results in repression of viral RNA synthesis [31]. However, our mass spectroscopy analysis showed phosphorylation of S308 in peptides from cells infected with rPIV5-W3:P(S157) and from cells infected with rPIV5-W3:P(F157) (although we could not quantitate the degree of phosphorylation at this, or other, residues which might be quite heterogeneous), and yet rPIV5-W3:P(F157) replication was not inhibited at late times p.i.. Furthermore, BI2536, an inhibitor of PLK1, did not influence the observed switch-off of PIV5-W3 protein synthesis nor did it prevent the establishment of persistence (S5 Fig). This strongly suggests that cellular kinases other than PLK1 are (also) responsible for phosphorylating P. Although, we do not know the other kinase(s) responsible, the fact that PIV5-W3 transcribes and replicates its genome efficiently until 12-18h p.i. suggests that they are unlikely to be constitutively expressed cellular kinases but may be induced kinases, for example the ER stress response kinases activated by PIV5 infection [44]. If the latter, then perhaps this may also be a mechanism that the virus has evolved to help establish persistence. We note that P is also highly phosphorylated in other paramyxoviruses (as are the phosphoproteins of other members of the order *Mononegavirales*, including Ebola virus*)* and that the level of phosphorylation influences viral transcription [28, 45, 46]. Furthermore, between different strains/isolates there can be substitutions of amino acids, including serine, that may be phosphorylated, opening up the possibility that lytic and persistent variants of these viruses may be selected *in vivo*.

Work presented here and elsewhere [31] clearly shows that phosphorylation of residues within the TSPPI motif (amino acids 155–159) and the NDS motif (amino acids 306–308) strongly influence the activity of the vRdRP. Residues 155–159 and 306–308 are outside the known N-terminal and C-terminal binding sites on P for NP [47, 48] and are outside its predicted oligomerisation domain [49]; no binding site for L has yet been mapped. It is interesting to note that a structural prediction for the P protein of the closely-related rubulavirus, PIV2 [50] places the 155–159 and 306–308 motifs in long non-structured regions that flank the predicted oligomerisation domain; it is tempting to speculate that phosphorylation alters the conformation of the non-structured regions thereby influencing the properties of the P protein. In this regard, it is of note that the threonine to proline substitution at residue 155 enhanced the vRdRP activity more than the serine to phenylalanine substitution at residue 157. Indeed, P155 led to the most active vRdRP in minigenome assays, even more active than A308 (and K306). The reason for this is unclear but suggests a model in which active and repressed states of vRdRP are in equilibrium, and that phosphorylation of P at residues 157 and 308 moves this equilibrium towards the repressed state. In this case, substituting threonine with proline at position 155 may cause a major structural change that locks vRdRP in the active state.

DVGs may also play a role in the establishment of persistent infections with PIV5, at least *in vitro*, as observed with other RNA viruses [38]. We show here that, with difficulty, persistently infected cell-lines can be established following a high moi of A549 cells with CPI+ lytic isolate of PIV5. In contrast to cells persistently infected with PIV5-W3, the CPI+ persistently infected cell-line had high levels of DVGs (S7 Fig), suggesting under these circumstances DVGs may play a role in CPI+ virus persistence. The characteristics of this persistently CPI+-infected cell-line was very different from that established by PIV5-W3. Ongoing virus transcription was much higher, the cells grew much more slowly and there were clear signs of a virus cytopathic effect. There was no evidence from HTS for selection of variants of CPI+ (e.g. S157) that would be predicted to have a persistence phenotype. However, this would be expected as the cells were initially infected at a high moi making it unlikely that such variants could be selected. Rather, as discussed above, we speculate that the selection of virus variants with a persistence phenotype, such as PIV5-W3, would likely occur following low moi infections *in vivo* in the presence of an ongoing adaptive immune response. Indeed, a critical point we are making here is that PIV5, and thus potentially other paramyxoviruses, may have evolved specific molecular mechanisms for the establishment of persistent infections which do not rely on the production of DVGs.

Although we have highlighted the importance of the phosphorylation status of P in determining whether or not a particular variant can establish persistence, it is possible that other single amino acid (nucleotide) changes in other genes, including L, may also play a role. We have also previously suggested that the interferon response may play an important role in repressing viral replication in some cells, thereby facilitating the establishment of persistence, and that there may be alternating selection of IFN-resistant and IFN-sensitive viruses during the acute and persistent phases of infection [11]. Interestingly, single amino acid (nucleotide) substitutions in the V protein, which is the viral IFN antagonist, can also determine whether a variant is IFN-sensitive or IFN-resistant [51]. Given that P and V are encoded by the same gene and share their N-terminal sequences, this gene may have evolved in such a manner as to facilitate the establishment of persistent infections. In this regard, it is of interest that PIV5 and other paramyxoviruses block the IFN response in such a way as not to cause cell death, which is a pre-requisite for establishing persistence.

PIV5 has been isolated on numerous occasions from a variety of host species but its association with disease is often tentative and unclear. Of possible relevance is that the disease potential of lytic and persistent variants of PIV5 is likely to be different. Thus, lytic variants may cause more cell death and spread more rapidly *in vivo* than persistent variants. Although PIV5 does not replicate to high titres in, or naturally infect, mice, this idea is supported by the observation that, rPIV5-W3:P(F157) replicated better than rPIV5-W3:P(S157) in mice and induced more cellular infiltration into the lungs of infected mice, which is a clear sign of greater pathology. Also if there are mixed populations of persistent and lytic variants *in vivo*, then the balance of the two may also influence disease outcomes. Interestingly, both lytic and persistent variants were detected by HTS in our stocks of PIV5-H221, which was isolated from a dog with kennel cough, but which had only been passaged a limited number of times in tissue culture cells following its initial isolation [13]. Thus, although the consensus sequence at position 157 of P was serine, phenylalanine was predicted in 4% of the viral population, and although the consensus at position 306 was a lysine, 5% of the sequenced population encoded asparagine [13].

It is of note that all the PIV5 strains sequenced, apart from the W3 strain, are predicted to have a lytic phenotype, which argues against the suggestion that viral transcription and replication are reduced at late times p.i. in order to limit the production of viral PAMPs and hence the induction of antiviral cytokines [31]. However, as lytic strains are more likely to induce a cytopathic effect than persistent variants, their selection may be favoured during clinical isolation. Furthermore, lytic variants, which give an obvious cytopathic effect in tissue culture cells, may evolve during the isolation of PIV5 from clinical material. This may have occurred during the isolation of PIV5 from human bone marrow cells, which were co-cultured with either MRC5 or Vero cells, as immunofluorescence was initially used to detect PIV5 during virus isolation as there was often an absence of a clear virus induced cytopathic effect [24, 52]. Similarly, during the isolation of cryptovirus (a strain of PIV5), human lymphocytes from a patient with SSPE (there is no suggestion that PIV5 can cause SSPE) were cultured with AV3 (continuous human amnion) cells, but the first clear signs of cytopathic effect only became visible after 20 passages [26]. On the other hand, tissue culture cell-lines can be persistently infected with PIV5 with no overt signs of infection, for example AGS cells which are commercially available from ATCC and ECACC [21].

Understanding the mechanisms by which paramyxoviruses, and other RNA viruses, can establish persistence *in vivo* is important for both fundamental and practical reasons. It may lead to a more complete view of viral epidemiology, and thus to better control measures. In addition, if the induction of long-lasting immunity is enhanced by viral persistence, then understanding the mechanisms by which viruses can establish such infections may lead to improved vaccine design.

## Materials and methods

### Cells and viruses

Vero, 293 and A549 cells (all from the European Collection of Authenticated Cell Cultures; ECACC) and derivatives were grown at 37°C as monolayers in 25 cm^2^ or 75 cm^2^ cell culture flasks, in Dulbecco's modified Eagle's medium (DMEM) supplemented with 10% (v/v) foetal bovine serum at 37°C. Stocks of PIV5 strains W3, LN, MEL, H221, SER and CPI+ (described in [13]) were grown and titrated in Vero cells. Commercial cell-viability assays (PrestoBlue (ThermoFisher Scientific) were performed according to manufacturer instructions.

### Preparation of [^35^S]-L-methionine-labelled total-cell extracts and SDS-PAGE

Infected or uninfected cells were metabolically labelled for 1h with [^35^S]-L-methionine (500Ci/mmol, MP Biomedical, USA) at various times p.i. as indicated in the text. After labelling, cells were lysed in disruption buffer, sonicated and heated for 5 min at 100°C and proteins were analysed by sodium dodecyl sulphate-polyacrylamide gel electrophoresis (SDS-PAGE). The gels were fixed, stained, dried, and resolved radiolabelled bands visualized by phosphorimager analysis.

### Fluorescence, immunoblotting, immunoprecipitation, immunostaining of virus plaques and FACS analysis

Procedures for immunoprecipitation, immunoblotting and immunofluorescence have been described previously [53, 54]. The antibodies used included monoclonal antibodies (mAbs) against PIV5 HN, P and NP [55] and against cellular MxA and β-actin (Sigma, A5441). Sialic acid on the surface of cells was visualised by staining with a recombinant protein in which green fluorescent protein (GFP) had been fused to two carbohydrate-binding modules derived from *Vibrio cholerae* [56]. Viral plaques were immunostained with a pool of mAbs against PIV5 followed by alkaline phosphatase-conjugated goat anti-mouse immunoglobulin G (Abcam, ab97020), and plaques were visualised with SigmaFast BCIP/NBT. For FAC-sorting, cells were prepared as a single-cell suspension by trypsinisation and immunostained with a pool of mAbs against HN. Single cells were sorted into individual wells of 96-well microtiter plates on the basis of whether or not they were positive for HN using a Becton Dickinson FACSJazz instrument.

### Generation of recombinant viruses

The changes in the P gene of the PIV5 W3 genome [T155P, S157F, K254R, N306K and S308A] were generated by primer-mediated mutagenesis using oligonucleotides purchased from Sigma and the modified fragments inserted into the rPIV5-W3 backbone plasmid, pBH276 [57] using standard molecular biology approaches. Base changes were confirmed by DNA sequencing. The pBH276-derived template plasmids (1μg) were transfected together with pCAGGS-based helper plasmids directing the synthesis of PIV5-NP (100ng), PIV5-P (100ng) and PIV5-L (500ng) into 6-well dishes containing ~10^6^ BSRT7 cells per well using linear polyethyleneimine (PEI) of molecular weight 25,000 (Polysciences Inc., Warrington PA, USA), or Fugene, under standard conditions. Successful recovery was confirmed by immunofluorescence screening using a monoclonal antibody (PIV5-Pk) conjugated to a FITC fluorophore, which recognises PIV5 V and P [55]. Working stocks of virus were produced from positive wells by two successive passages at low multiplicity of infection (moi) in Vero cells, and stocks were harvested, clarified by centrifugation, and flash frozen in liquid nitrogen.

### Identification of phosphorylation sites on P

Confluent monolayers of A549 cells, grown in 25 cm^2^ flasks, were infected at a high moi with either rPIV5-W3:P(S157) or rPIV5-W3:P(F157) and at 24h p.i. were lysed in disruption buffer and submitted to the FingerPrints Proteomics Facility (University of Dundee, UK) for SDS-PAGE gel analysis. The samples were run on a 4–12 Bis-Tris gel with MOPS running buffer (Thermo Fisher Scientific) and the gel stained with SimplyBlue SafeStain (Thermo Fisher Scientific). PIV5 P bands (~45kDa) were excised for in-gel processing and trypsin digestion prior to analysis by mass spectrometry using a RSLCnano UHPLC system coupled to a LTQ Orbitrap Velos Pro mass spectrometer (Thermo Scientific). The resultant data were analysed using the Mascot Search engine (Version 2.4.1) using the Sprot Human database and the sites of phosphorylation annotated using the Mascot delta score.

### High throughput nucleotide sequencing

Infected cells in 25cm^2^ flasks were lysed in 1 ml of Trizol and RNA was extracted using a Direct-zol RNA miniprep kit (Zymo). A directional sequencing library was prepared from rRNA-depleted RNA using a TruSeq stranded total RNA library prep kit (Illumina, U.K.). Quality control and quantification of the cDNA library were monitored using DNA-specific 1000 or 5000 chips on a Bioanalyzer 2100 (Agilent Technologies) and a Qubit fluorometer (Invitrogen). Individual libraries were pooled at 10 nM each and sequenced on the MiSeq platform (Illumina). Abundances of genome and antigenome/mRNA reads were calculated relative to total read numbers (including cellular reads) from which residual rRNA and mitochondrial RNA reads had been removed. These reads were identified by aligning the trimmed, filtered data to reference genomes for human 18S, 28S, 5S and 5.8S rRNA and mitochondrial DNA (accession numbers NR_003286.2, NR_003287, X51545, J01866, NC_012920), and then removed.

The presence of defective virus genomes (DVGs) was assessed using ViReMa (Routh and Johnson 2013). ViReMa detects potential recombination by identifying reads that contain sequences mapping to different regions of the genome, and thus facilitates the identification and quantification of DVG populations.

### Minigenome assays

Using standard techniques the *Renilla* luciferase gene in pSMG-RL [36] was replaced by a gene encoding firefly luciferase. An additional modification to reduce transcriptional readthrough from cryptic promoters within the vector backbone was made by incorporating two copies of a 237 bp fragment from SV40 (coordinates 2533–2770) that includes the bidirectional polyadenylation site and transcriptional terminator site downstream from the T7 RNA polymerase terminator sequence. To determine the activity of the minigenome, 25ng of the resulting plasmid (pPIV5MG-Fluc.ter), was transfected into 293 cells together with pCAGGS-based helper plasmids directing the synthesis of PIV5-NP (100ng), PIV5-P (100ng) and PIV5-L (500ng), 500ng of a pCAGGS-based plasmid directing the synthesis of T7 RNA polymerase (codon-optimised for expression in human cells), and 50ng of a β-galactosidase-expressing transfection control plasmid, pCATlac. Transient transfections used PEI and were left for 40 h before harvesting. Luciferase and β-galactosidase activity assays were carried out and normalised as previously described [58]. Variants of the P gene were generated by primer-mediated mutagenesis as described above.

### Infection of mice

Female BALB/c mice were obtained from Charles River (Bath) at 7–9 weeks of age and housed in accordance with the United Kingdom Home Office guidelines. All work was conducted with approval from the Animal Welfare and Ethical Review Board of Imperial College London. Mice were infected intranasally with 2 x 10^6^ plaque-forming units (pfu) of virus in 100 μl. Mice were provided with food and water *ad libitum* and monitored daily for signs of illness. Statistical comparisons of mouse data were as described in Figure legends were performed using Prism 6 (GraphPad Software Inc., La Jolla, CA, USA).

### PIV5 load measurements in mice

Viral load in lung tissue was assessed by quantitative PCR (qPCR) of bulk PIV5 M gene RNA. RNA was extracted from frozen lung tissue using Trizol extraction after homogenisation in a TissueLyzer (Qiagen) and converted into cDNA using random primers (GoScript, Promega). qPCR of PIV5 M gene was carried out using SYBRselect master mix and 250 nM forward (5’-TCATGAGCCACTGGTGACAT-3’) and reverse (5’-TGGAATTCCCTCAGTTGTCC-3’) primers on a Stratagene Mx3005p instrument (Agilent Technologies). In order to normalise M gene levels, levels of cellular *Gapdh* mRNA were measured using forward (5’-AGGTCGGTGTGAACGGATTTG-3’) and reverse (5’-TGTAGACCATGTAGTTGAGGTCA-3’) primers.

### Lung cell isolation from mice

Lung tissue was homogenised through a 100μm cell strainer and centrifuged at 500 x g for 5 minutes, as described previously [59]. Supernatants were removed, and red blood cell lysis buffer (ACK lysing buffer, ThermoFisher) was added to the cell pellet and mixed for 5 min before a further centrifugation at 500 x g for 5 minutes. Remaining cells were resuspended in DMEM and viable numbers were quantified by trypan blue exclusion.

### Ethics statement

All animal experiments were performed in accordance with the United Kingdom’s Home Office guidelines under PPL P4EE85DED and all work was approved by the Animal Welfare and Ethical Review board (AWERB) at Imperial College London. Studies followed the ARRIVE guidelines and all animal infections and infectious work was carried out in biosafety level two facilities.

## Supporting information

S1 Fig The majority of A549 cells do not die and become persistently infected following high moi infections with PIV5-W3. Monolayers of A549 cells were either mock infected or infected with PIV5-W3 at 10 pfu/cell and at 24 and 96 h p.i. were fixed and immunostained with an anti-NP monoclonal antibody. Phase contrast images of the monolayers prior to fixing and staining are also shown.
(TIF)

S2 Fig A decrease in the synthesis of all viral proteins in A549 cells infected with PIV5-W3 is observed by 36h p.i. Monolayers of A549 cells were either mock infected or infected with PIV5-W3 at 10 pfu/cell and at the times indicated the cells were metabolically labelled for 1h with [^35^S]-L-methionine and the viral proteins immune-precipitated. Total cell extracts (left-hand panels) and immune precipitates (right-hand panels) were separated by electrophoresis through a 4–12% SDS-PAG; the total protein content of the samples was visualised by staining the gels with Coomassie Brilliant Blue and labelled proteins visualized using a phosphoimager. The positions that the NP and M polypeptides migrate to in the total cell extracts are indicated by asterisks as are the positions of the immunoglobulin heavy (IgH) and light (IgL) chains.
(TIF)

S3 Fig PIV5-W3 protein synthesis is repressed with time p.i. in cells unable to produce IFN. In parallel to the experiment shown in Fig 1, panel a, monolayers of A549/BVDV-Npro cells were either mock-infected or infected with PIV5-W3 at 10 pfu/cell in the presence or absence of Ruxolitinib (2μg/ml). At the times indicated the cells were metabolically labelled for 1h with [^35^S]-L-methionine. Polypeptides present in total cell extracts were separated by electrophoresis through a 4–12% SDS-PAG, and the labelled polypeptides visualized using a phosphorimager. The positions of the NP and M polypeptides are indicated by asterisks.
(TIF)

S4 Fig Mass spectroscopy was used to map the phosphorylation sites on P of rPIV5-W3:P(S157) and rPIV5-W3:P(F157). Amino acids which were confidently identified as being phosphorylated are highlighted in red; those that had a level of ambiguity are highlighted blue. Amino acid residue numbers are indicated at the right-hand side of the Figure and the serine residues at positions 157 and 308 have been highlighted by a dark orange box.
(TIF)

S5 Fig Inhibition of PLK1 by BI 2536 did not significantly affect the kinetics of PIV5-W3 protein synthesis inhibition. Monolayers of A549 cells were either mock infected or infected with rPIV5-W3:P(S157) or CPI+ at 10 pfu/cell, in the presence or absence of the PLK1 inhibitor BI 2536 (1μM). At the times indicated cells were metabolically labelled for 1h with [^35^S]-L-methionine. Polypeptides present in the total cell extracts were separated by electrophoresis through a 4–12% SDS-PAG, and the labelled polypeptides visualized using a phosphorimager. 1μM of BI 2536 completely inhibited the progression through mitosis of parallel cultures of mock-infected cells as shown by the lack of mitotic cells after staining the cells with DAPI and as described in [1]. The positions that the NP and M polypeptides migrate to in the total cell extracts are indicated by asterisks.
(TIF)

S6 Fig **Panel a) Transcription of PIV5-CPI+ mRNA synthesis is not inhibited at late times p.i. Monolayers of A549 cells grown in 25cm flasks were infected with PIV5-CPI+ at 10 pfu/cell, RNA was extracted at 6, 12, 18, 24, and 48 p.i. (by 96h p.i. the majority of cells had died) and subjected to total RNA sequencing following rRNA and mitochondrial RNA reduction.** Directional sequence analysis was performed, and the percentage of viral mRNA and genome reads were compared to the cellular reads at each time point. Panel b) Viral mRNA synthesis in cells infected with rPIV5-W3:P(F157) is significantly higher than in cells infected with rPIV5-W3:P(S157). A549 cells were infected with rPIV5-W3:P(S157) or rPIV5-W3:P(F157) at 10 pfu/cell and RNA was extracted at 24 p.i. then subjected to total RNA sequencing as described above. The bars show standard deviation values based on three samples for PIV5-W3:P(S157)-infected cells (the same as those shown in Fig 2), two samples for rPIV5-W3:P(F157)-infected cells. Note that although only 1 CPI+ sample for each time point was analysed the percentage of viral mRNA to total cellular mRNA at 18, 24 and 48h p.i. was very similar.
(TIF)

S7 Fig Defective viral genomes (DVGs) cannot be detected in A549 cells persistently infected with PIV5-W3 but are present in cells persistently infected with CPI+. To determine whether HTS could be employed to detect the presence of DVGs in persistently infected cells, with or without the need for prior nucleocapsid purification, A549 cells were infected with a DVG-rich stock of PIV5-W3(VΔC) [2] at 10 pfu/cell. At 24 h p.i., RNA was extracted either directly from the infected cells or from viral nucleocapsids (NC) purified on a CsCl gradient as described in [2]. The total cell (TC) RNA preparations were subjected to ribosomal RNA (rRNA) and mitochondrial RNA reduction and, together with the RNA extracted from the purified nucleocapsids, sequenced directionally. The data were subjected to analysis using ViReMa in order to identify breakpoints at which the vRdRP had effectively jumped along the template to produce an internal deletion DVG or, alternatively, switched to the nascent strand to generate a copyback DVG. In agreement with the results of Killip et al [2], no internal deletion DVGs were detected in either RNA preparation, but several distinct populations of copyback DVGs were identified in both (Table A). Importantly, there was no significant difference between the results obtained from the two RNA samples, and six identical DVG populations were identified in both in almost identical proportions. The sequence which contained breakpoint reads of the lowest copyback DVG detected contributed only 0.02% of the total genomic RNA reads. To estimate of the ratio of DVGs to non-defective virus genomes the average number of reads per nucleotides (nt) from a region of the genome that was common to all the DVGs (15162–15230: X) minus the average number of reads per nt prior to the first identified breakpoint (1–13999: Y) was divided by the average number of reads per nt prior to the first identified breakpoint (1–13999: Y), i.e. X-Y/Y (Table A).
Using the same approach, no internal or copy back DVGs could be detected in nucleocapsid purified RNA or total cell RNA isolated from passage 3 PIV5-W3 persistently infected cells. In contrast, high levels of copyback DVGs could be detected in nucleocapsids purified isolated from cells persistently infected with CPI+ (Table B; total cell RNA was not sequenced). These results showed that the total ratio of DVGs to non-defective genomes was 0.8:1. Also, as would be expected given that the template switch that leads to the generation of DVGs is thought to be random, the breakpoint position for the CPI+ DVGs is different from those present in PIV5-W3(VΔC) preparations.
(TIF)
