# Supplementary figures and images for: The switch between acute and persistent paramyxovirus infection caused by single amino acid substitutions in the RNA polymerase P subunit
Source: PLoS Pathog. 2019 Feb 11;15(2):e1007561. doi: 10.1371/journal.ppat.1007561 (PMC6386407; doi:10.1371/journal.ppat.1007561)

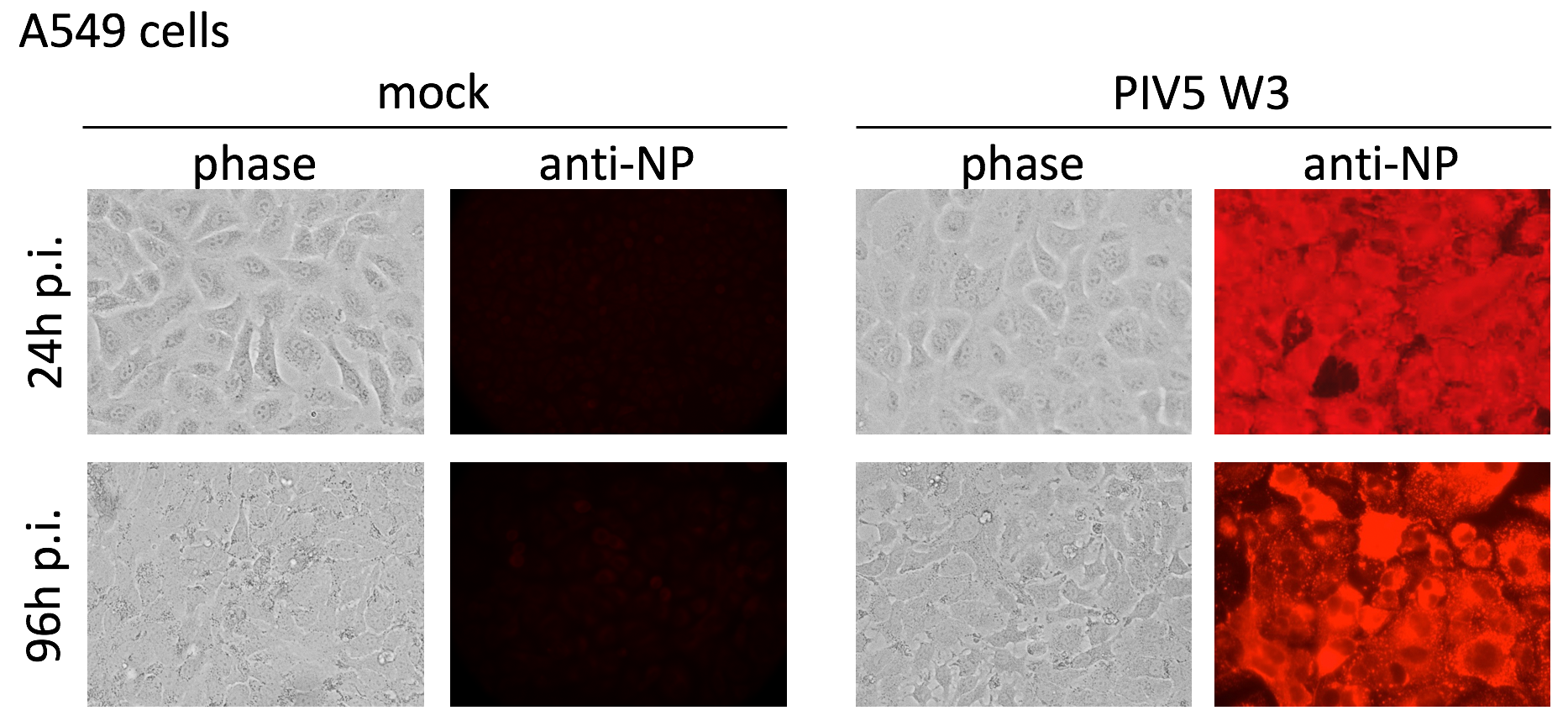

Supplement: S1 Fig — Monolayers of A549 cells were either mock infected or infected with PIV5-W3 at 10 pfu/cell and at 24 and 96 h p.i. were fixed and immunostained with an anti-NP monoclonal antibody. Phase contrast images of the monolayers prior to fixing and staining are also shown. (TIF) [file ppat.1007561.s001.tif]

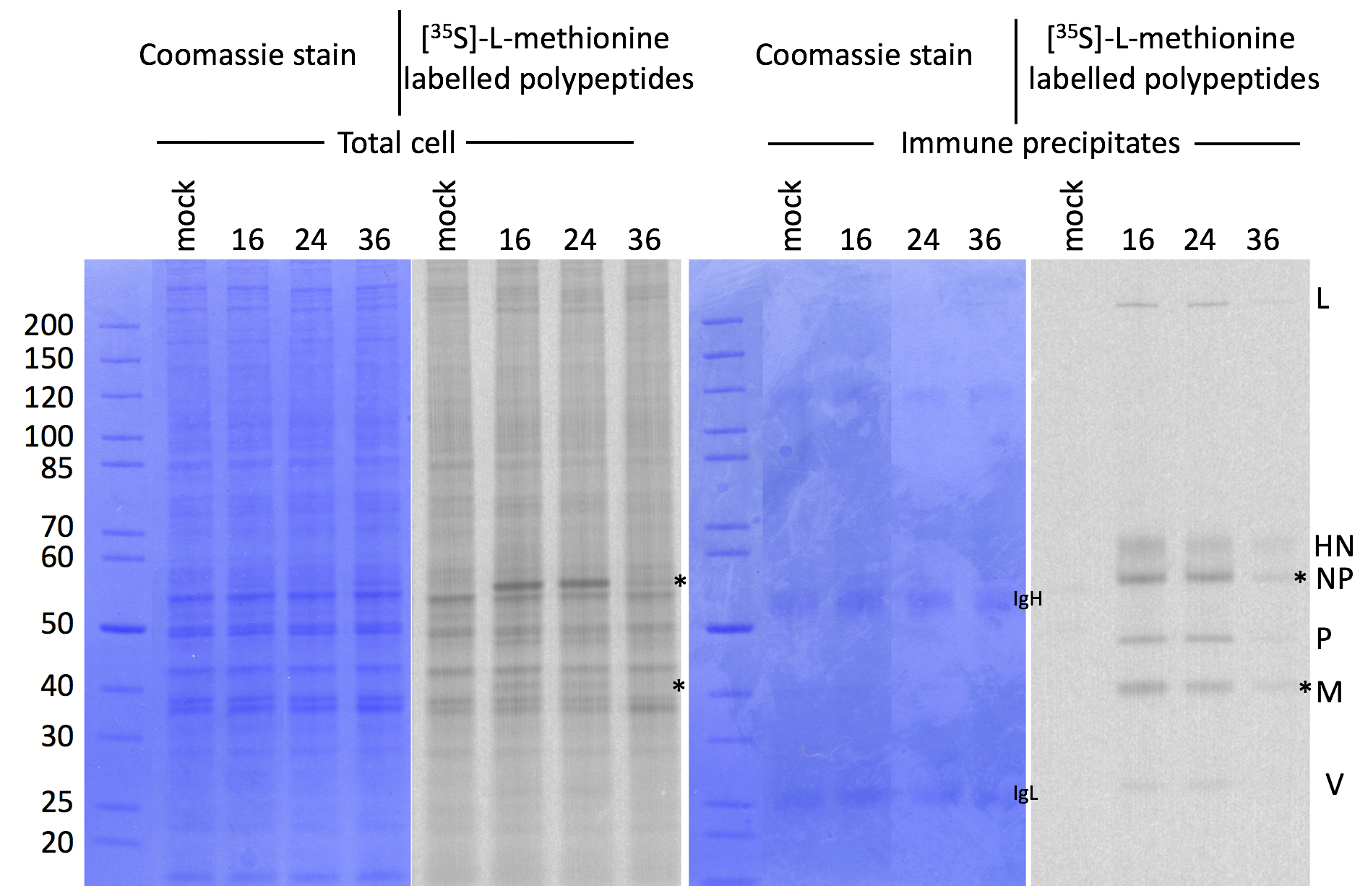

Supplement: S2 Fig — Monolayers of A549 cells were either mock infected or infected with PIV5-W3 at 10 pfu/cell and at the times indicated the cells were metabolically labelled for 1h with [35S]-L-methionine and the viral proteins immune-precipitated. Total cell extracts (left-hand panels) and immune precipitates (right-hand panels) were separated by electrophoresis through a 4–12% SDS-PAG; the total protein content of the samples was visualised by staining the gels with Coomassie Brilliant Blue and labelled proteins visualized using a phosphoimager. The positions that the NP and M polypeptides migrate to in the total cell extracts are indicated by asterisks as are the positions of the immunoglobulin heavy (IgH) and light (IgL) chains. (TIF) [file ppat.1007561.s002.tif]

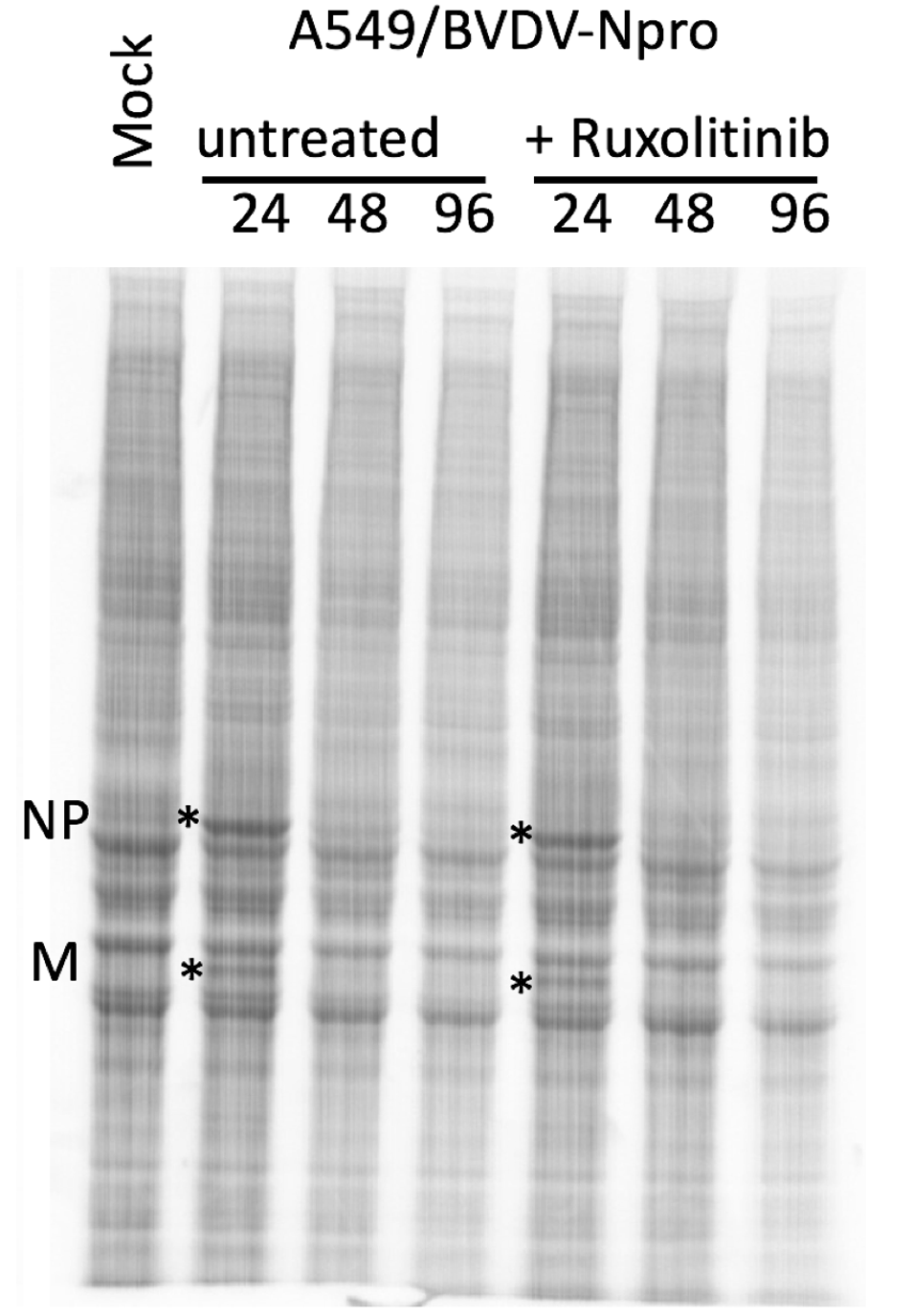

Supplement: S3 Fig — In parallel to the experiment shown in Fig 1, panel a, monolayers of A549/BVDV-Npro cells were either mock-infected or infected with PIV5-W3 at 10 pfu/cell in the presence or absence of Ruxolitinib (2μg/ml). At the times indicated the cells were metabolically labelled for 1h with [35S]-L-methionine. Polypeptides present in total cell extracts were separated by electrophoresis through a 4–12% SDS-PAG, and the labelled polypeptides visualized using a phosphorimager. The positions of the NP and M polypeptides are indicated by asterisks. (TIF) [file ppat.1007561.s003.tif]

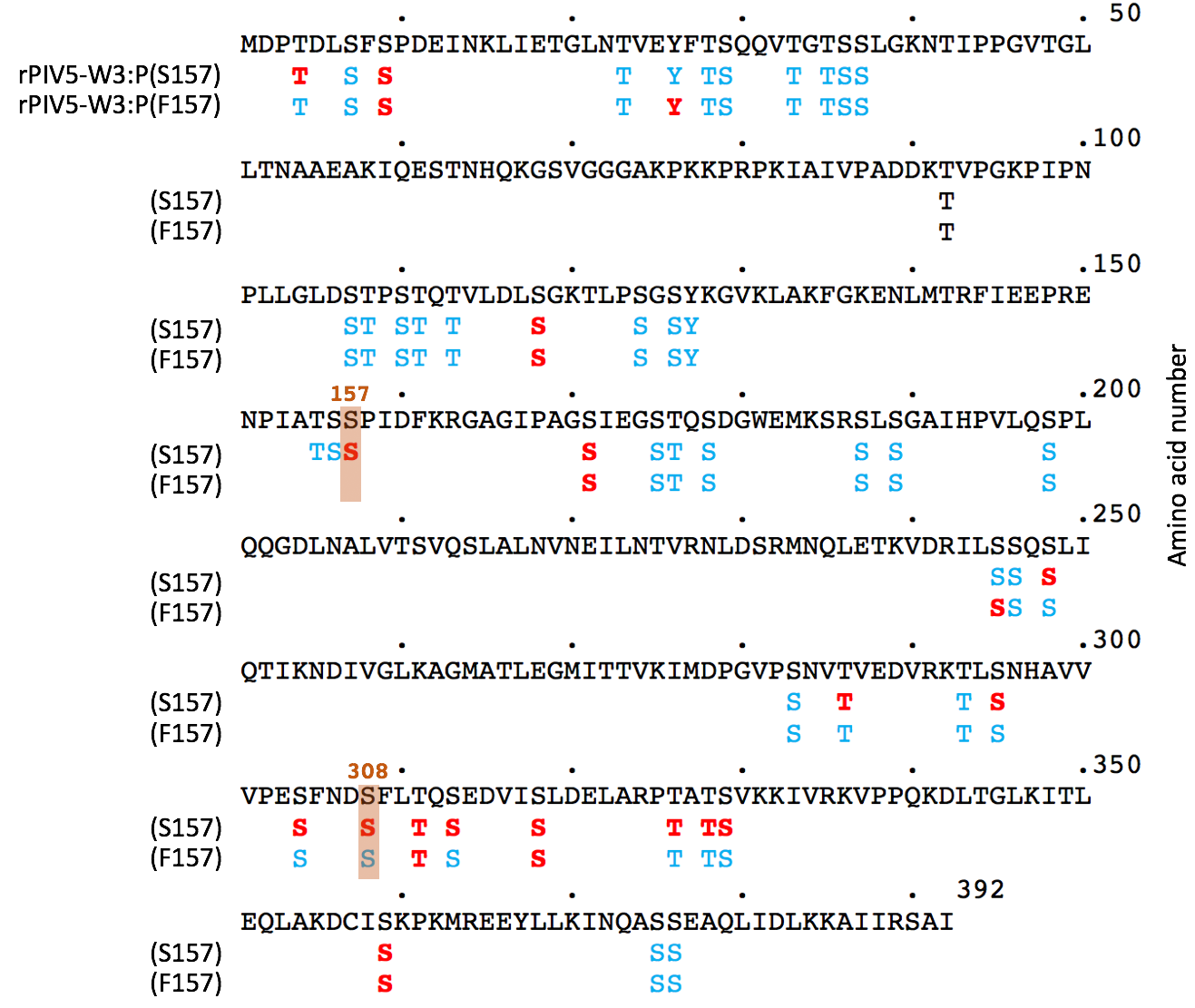

Supplement: S4 Fig — Amino acids which were confidently identified as being phosphorylated are highlighted in red; those that had a level of ambiguity are highlighted blue. Amino acid residue numbers are indicated at the right-hand side of the Figure and the serine residues at positions 157 and 308 have been highlighted by a dark orange box. (TIF) [file ppat.1007561.s004.tif]

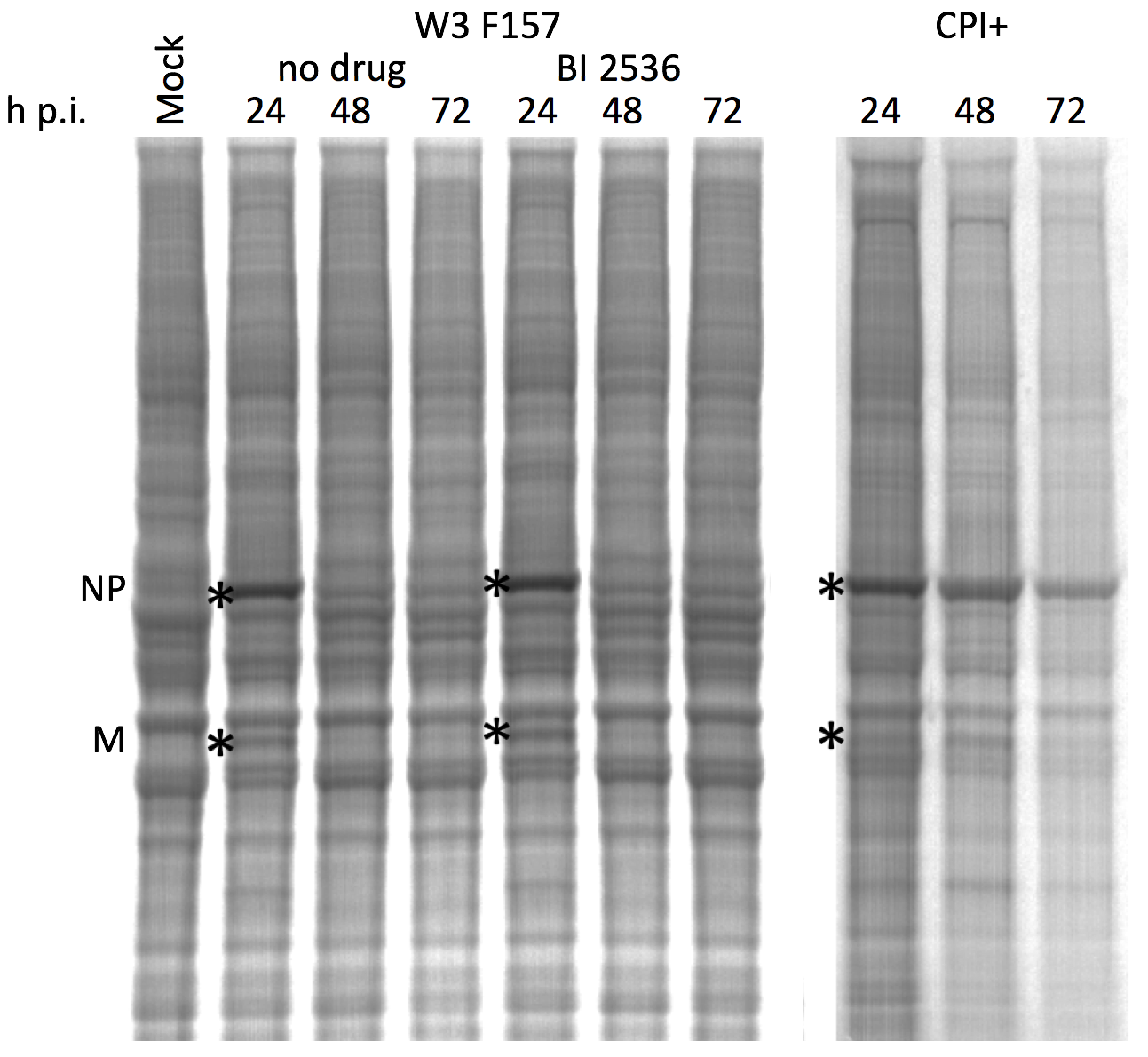

Supplement: S5 Fig — Monolayers of A549 cells were either mock infected or infected with rPIV5-W3:P(S157) or CPI+ at 10 pfu/cell, in the presence or absence of the PLK1 inhibitor BI 2536 (1μM). At the times indicated cells were metabolically labelled for 1h with [35S]-L-methionine. Polypeptides present in the total cell extracts were separated by electrophoresis through a 4–12% SDS-PAG, and the labelled polypeptides visualized using a phosphorimager. 1μM of BI 2536 completely inhibited the progression through mitosis of parallel cultures of mock-infected cells as shown by the lack of mitotic cells after staining the cells with DAPI and as described in [1]. The positions that the NP and M polypeptides migrate to in the total cell extracts are indicated by asterisks. (TIF) [file ppat.1007561.s005.tif]

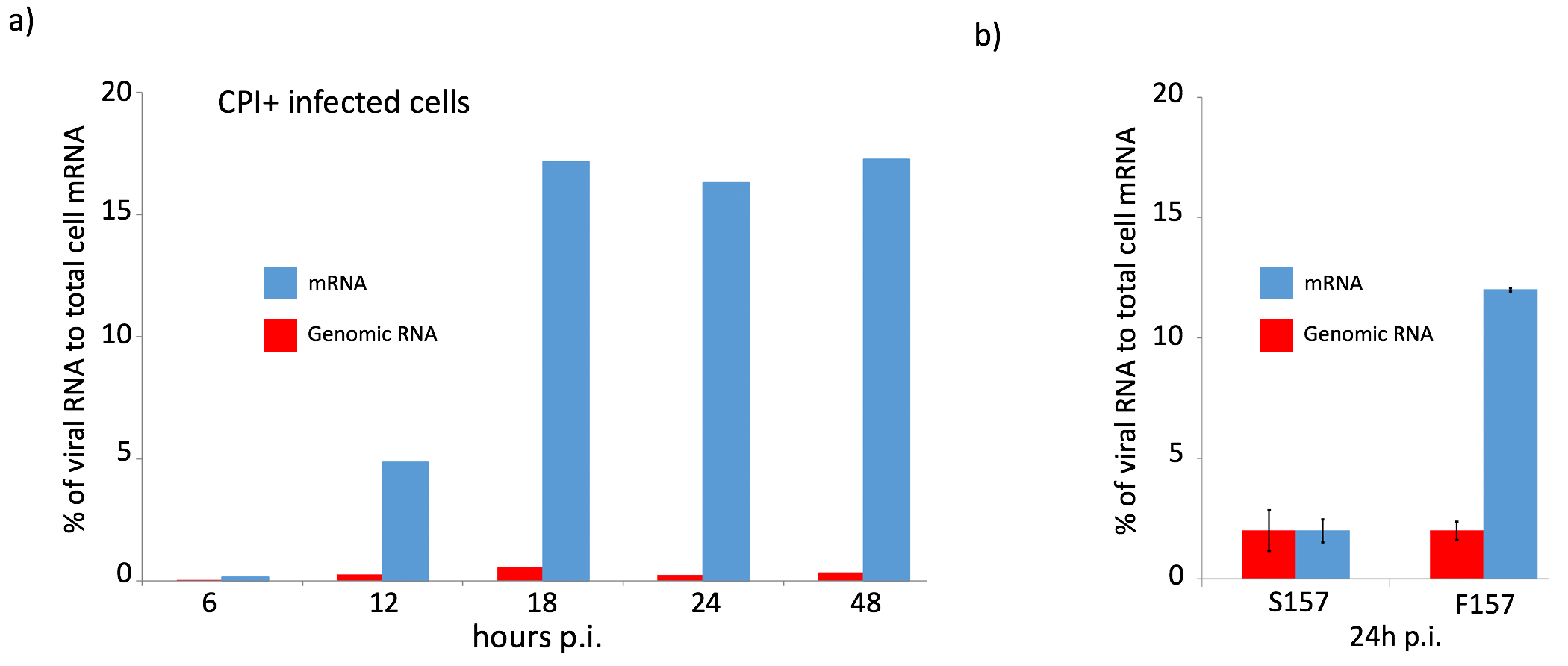

Supplement: S6 Fig — Panel a) Transcription of PIV5-CPI+ mRNA synthesis is not inhibited at late times p.i. Monolayers of A549 cells grown in 25cm flasks were infected with PIV5-CPI+ at 10 pfu/cell, RNA was extracted at 6, 12, 18, 24, and 48 p.i. (by 96h p.i. the majority of cells had died) and subjected to total RNA sequencing following rRNA and mitochondrial RNA reduction. Directional sequence analysis was performed, and the percentage of viral mRNA and genome reads were compared to the cellular reads at each time point. Panel b) Viral mRNA synthesis in cells infected with rPIV5-W3:P(F157) is significantly higher than in cells infected with rPIV5-W3:P(S157). A549 cells were infected with rPIV5-W3:P(S157) or rPIV5-W3:P(F157) at 10 pfu/cell and RNA was extracted at 24 p.i. then subjected to total RNA sequencing as described above. The bars show standard deviation values based on three samples for PIV5-W3:P(S157)-infected cells (the same as those shown in Fig 2), two samples for rPIV5-W3:P(F157)-infected cells. Note that although only 1 CPI+ sample for each time point was analysed the percentage of viral mRNA to total cellular mRNA at 18, 24 and 48h p.i. was very similar. (TIF) [file ppat.1007561.s006.tif]

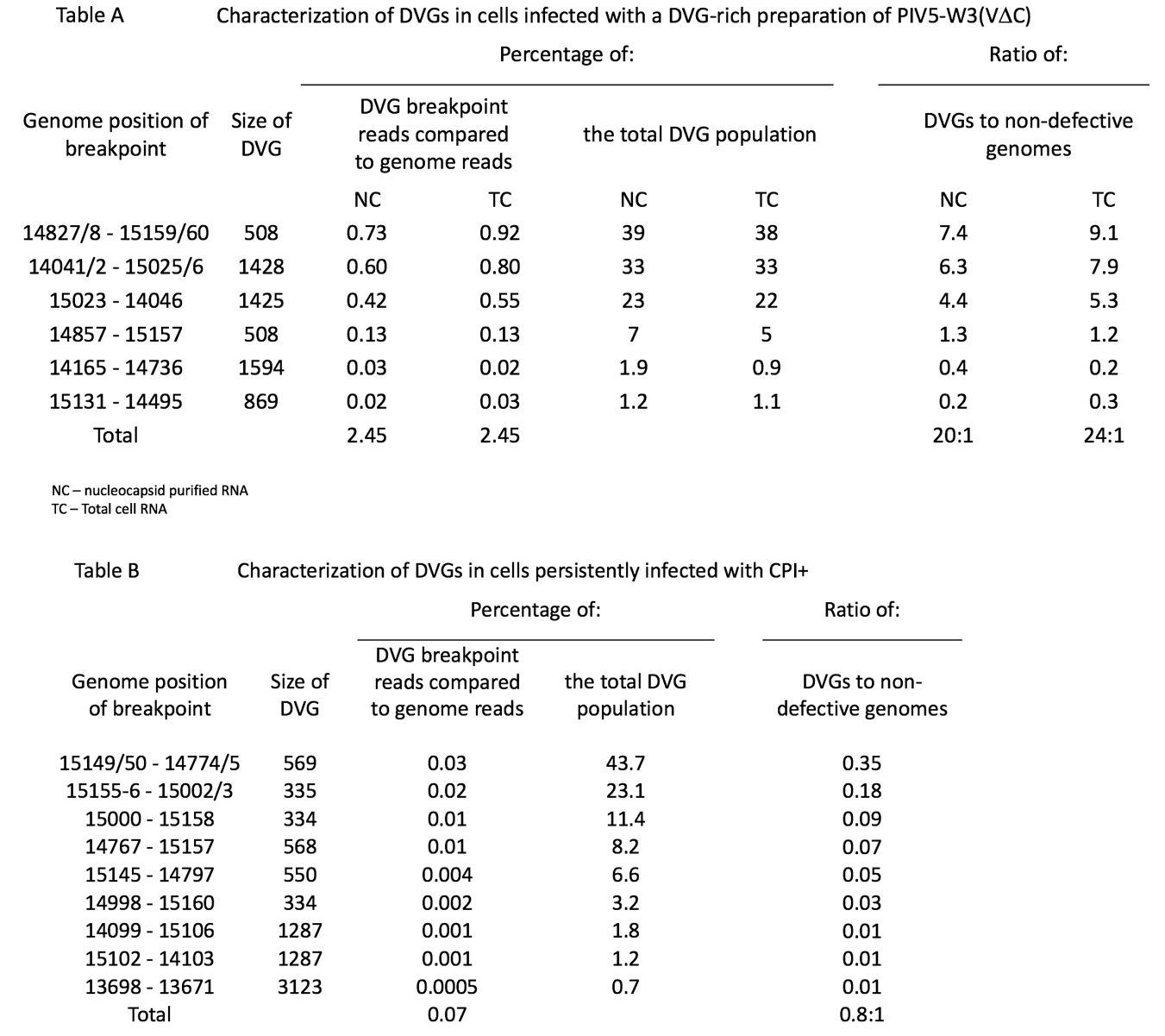

Supplement: S7 Fig — To determine whether HTS could be employed to detect the presence of DVGs in persistently infected cells, with or without the need for prior nucleocapsid purification, A549 cells were infected with a DVG-rich stock of PIV5-W3(VΔC) [2] at 10 pfu/cell. At 24 h p.i., RNA was extracted either directly from the infected cells or from viral nucleocapsids (NC) purified on a CsCl gradient as described in [2]. The total cell (TC) RNA preparations were subjected to ribosomal RNA (rRNA) and mitochondrial RNA reduction and, together with the RNA extracted from the purified nucleocapsids, sequenced directionally. The data were subjected to analysis using ViReMa in order to identify breakpoints at which the vRdRP had effectively jumped along the template to produce an internal deletion DVG or, alternatively, switched to the nascent strand to generate a copyback DVG. In agreement with the results of Killip et al [2], no internal deletion DVGs were detected in either RNA preparation, but several distinct populations of copyback DVGs were identified in both (Table A). Importantly, there was no significant difference between the results obtained from the two RNA samples, and six identical DVG populations were identified in both in almost identical proportions. The sequence which contained breakpoint reads of the lowest copyback DVG detected contributed only 0.02% of the total genomic RNA reads. To estimate of the ratio of DVGs to non-defective virus genomes the average number of reads per nucleotides (nt) from a region of the genome that was common to all the DVGs (15162–15230: X) minus the average number of reads per nt prior to the first identified breakpoint (1–13999: Y) was divided by the average number of reads per nt prior to the first identified breakpoint (1–13999: Y), i.e. X-Y/Y (Table A). Using the same approach, no internal or copy back DVGs could be detected in nucleocapsid purified RNA or total cell RNA isolated from passage 3 PIV5-W3 persistently infected cells. In cont [file ppat.1007561.s007.tif]
